# RRM2 and CDC6 are novel effectors of XBP1-mediated endocrine resistance and predictive markers of tamoxifen sensitivity

**DOI:** 10.1186/s12885-023-10745-1

**Published:** 2023-03-30

**Authors:** David Barua, Afrin Sultana, Md Nahidul Islam, Fergus Cox, Ananya Gupta, Sanjeev Gupta

**Affiliations:** 1 Discipline of Pathology, Cancer Progression and Treatment Research Group, Lambe Institute for Translational Research, School of Medicine, University of Galway, Galway, Ireland; 2 Discipline of Biochemistry, School of Medicine, University of Galway, Galway, Ireland; 3 Discipline of Physiology, Human Biology Building, School of Medicine, University of Galway, Galway, Ireland

**Keywords:** XBP1, Endocrine resistance, RRM2, CDC6, Breast cancer, ER stress

## Abstract

**Background:**

Endocrine-resistant breast cancers have elevated expression of XBP1, where it drives endocrine resistance by controlling the expression of its target genes. Despite the in-depth understanding of the biological functions of XBP1 in ER-positive breast cancer, effectors of endocrine resistance downstream of XBP1 are poorly understood. The aim of this study was to identify the XBP1-regulated genes contributing to endocrine resistance in breast cancer.

**Methods:**

XBP1 deficient sub-clones in MCF7 cells were generated using the CRISPR-Cas9 gene knockout strategy and were validated using western blot and RT-PCR. Cell viability and cell proliferation were evaluated using the MTS assay and colony formation assay, respectively. Cell death and cell cycle analysis were determined using flow cytometry. Transcriptomic data was analysed to identify XBP1-regulated targets and differential expression of target genes was evaluated using western blot and qRT-PCR. Lentivirus and retrovirus transfection were used to generate RRM2 and CDC6 overexpressing clones, respectively. The prognostic value of the XBP1-gene signature was analysed using Kaplan–Meier survival analysis.

**Results:**

Deletion of XBP1 compromised the upregulation of UPR-target genes during conditions of endoplasmic reticulum (EnR) stress and sensitized cells to EnR stress-induced cell death. Loss of XBP1 in MCF7 cells decreased cell growth, attenuated the induction of estrogen-responsive genes and sensitized them to anti-estrogen agents. The expression of cell cycle associated genes RRM2, CDC6, and TOP2A was significantly reduced upon XBP1 deletion/inhibition in several ER-positive breast cancer cells. Expression of RRM2, CDC6, and TOP2A was increased upon estrogen stimulation and in cells harbouring point-mutants (Y537S, D538G) of ESR1 in steroid free conditions. Ectopic expression of RRM2 and CDC6 increased cell growth and reversed the hypersensitivity of XBP1 KO cells towards tamoxifen conferring endocrine resistance. Importantly, increased expression of XBP1-gene signature was associated with poor outcome and reduced efficacy of tamoxifen treatment in ER-positive breast cancer.

**Conclusions:**

Our results suggest that RRM2 and CDC6 downstream of XBP1 contribute to endocrine resistance in ER-positive breast cancer. XBP1-gene signature is associated with poor outcome and response to tamoxifen in ER-positive breast cancer.

**Supplementary Information:**

The online version contains supplementary material available at 10.1186/s12885-023-10745-1.

## Background

The majority of breast cancers express estrogen receptor α (ER) protein, and as such these tumours are treated with endocrine reagents that target the ER function [1]. The three main categories of hormonal therapies used to treat ER-positive breast cancer are (i) tamoxifen which act as selective estrogen receptor modulators (ii) fulvestrant which acts as selective estrogen receptor degrader, and (iii) letrozole and exemestane which reduce the production of endogenous estrogen [2]. Despite the robust efficacy of all these potent endocrine therapies, endocrine resistance develops over time, and eventually cancer relapses leading to disease progression and metastasis. Several studies have reported that unfolded protein response (UPR) is one of the crucial signalling node contributing to the development of endocrine resistance in ER-positive breast cancer [3]. Activation of UPR helps cancer cells to survive and proliferate during stressful conditions of tumour microenvironment such as hypoxia and nutrient deprivation, thereby contribute to therapy resistance [4]. The UPR is a signalling cascade initiated in response to endoplasmic reticulum (EnR) stress, caused by accumulation of misfolded and/or unfolded proteins in EnR. There are three EnR stress sensors present in the EnR membrane namely, (i) Double-stranded RNA-activated protein kinase-like ER kinases (PERK), (ii) Inositol requiring enzyme 1α (IRE1), and (iii) Activating transcription factor 6α (ATF6) [5]. The EnR resident chaperone, Glucose-regulated protein 78 (GRP78) binds to the luminal domain of PERK, IRE1, and ATF6 and keeps their activity under control. However, during the conditions of EnR stress GRP78 dissociates from these sensors, leading to their activation [6]. The main goal of UPR is to restore EnR homeostasis by reducing the load of client proteins entering the EnR and degradation of unfolded and/or misfolded proteins. However, during prolonged or severe EnR stress the UPR promotes apoptosis. Induction of cell survival signalling is directed through the IRE1-XBP1 axis, whereas the cell death activation during UPR is governed by PERK-CHOP signalling [5, 6].

X-box binding protein-1 (XBP1) constitutes a key signalling node of UPR whose expression is significantly increased in ER-positive breast cancer [3]. There are two distinct forms of XBP1, unspliced XBP1 (XBP1u) and spliced XBP1 (XBP1s). During the conditions of EnR stress, activated IRE1α catalyses the non-canonical splicing of XBP1u mRNA, excising 26 nucleotides that changes its reading frame resulting in 56 KD spliced XBP1 (XBP1s) protein production [7]. Unlike XBP1u mRNA, XBP1s encodes for active and stable transcription factor that upregulates the expression of genes involved in maintaining EnR homeostasis such as protein folding, glycosylation, degradation and protein secretion in almost all model systems [8]. In addition there is subset of genes whose expression is regulated by XBP1s in cell type- and stimuli-specific manner [8]. Several studies have reported crucial role of XBP1s in ER-positive as well as triple-negative breast cancer (TNBC) [9–11]. In TNBC, XBP1s forms a complex with HIF1α and upregulates the expression of hypoxia response pathway genes [9]. In ER-positive breast cancer XBP1s upregulates the expression of nuclear receptor coactivator 3 (NCOA3) via consensus XBP1-binding site in its promoter. Furthermore, NCOA3 is required for optimal induction of XBP1 expression upon estrogen stimulation, creating a positive feedback loop comprising XBP1s and NCOA3 [10]. Ectopic expression of XBP1s increases the expression of p65/RelA in ER-positive breast cancer [11]. XBP1s can contribute to endocrine resistance by physically interacting with ER and enhancing the transcriptional activity of ER [12]. However, molecular effectors of XBP1s that mediate development of endocrine resistance in ER-positive breast cancer are not fully understood.

In this study, we generated XBP1 KO sub-clones of MCF7 cells and confirmed its important role during conditions of EnR stress. Here, we show that loss of XBP1 sensitizes MCF7 cells specifically to EnR stress-induced cell death. We show that XBP1 is required for optimal cell growth, induction of estrogen-responsive genes and response towards anti-estrogens. Using genetic and chemical inhibition of XBP1 we show that RRM2, CDC6 and TOP2A are regulated by XBP1 as well as ER. We also show that ectopic expression of RRM2 and CDC6 can reverse the hypersensitivity of XBP1 KO cells towards tamoxifen. Increased expression of XBP1-gene signature is associated with poor outcome and resistance to tamoxifen in ER-positive breast cancer. Our results showing the increased expression of RRM2, CDC6 and TOP2A in XBP1-dependent manner provides a mechanism for their overexpression in breast cancers.

## Methods

### Cell culture and reagents

MCF7, T47D, and BT474 cells were procured from ECACC (Salisbury, Sussex, UK). MCF7 cells expressing Y537S and D538G mutants were kind gifts from Dr. Steffi Oesterreich (University of Pittsburgh, USA). HEK 293 T cells were obtained from the Indiana University National Gene Vector Biorepository (Indianapolis, IN, USA). Cells were maintained in DMEM supplemented with 10% foetal calf serum (FCS), 100 U/ml penicillin, and 100 mg/ml streptomycin at 37 °C with 5% CO_2_. MCF7 cells expressing Y537S and D538G mutants were cultured using DMEM and 5% FCS. FCS and charcoal-stripped serum (CSS) were purchased from Labtech International (Sussex, UK). Thapsigargin (TG), Tunicamycin (TM), STF083010, and Blasticidin were purchased from Tocris (Bio-Techne Ltd, Abingdon, UK). Tamoxifen, fulvestrant, and AZD9496 were purchased from MedChem Tronica, Sollentuna, Sweden. Pevonedistat (MLN4924) was from EMD Millipore, Wicklow, Ireland. All other chemicals were obtained from Sigma-Aldrich, Wicklow, Ireland, unless otherwise stated.

### Plasmid constructs

CRISPR guide RNA plasmids (Cat# KN201959) targeting XBP1 were purchased from Origene (Cambridge Biosciences, Cambridge, UK). The following plasmids: psPAX2 (Cat# 12,260) and PMD2.G (Cat# 12,259), PCLXSN (Cat# 12,343), CDC6 (Cat # 109,332 and 109,333), and pUMVC (Cat# 8449) were from Addgene, Watertown, MA, USA. Null control and V5-tagged RRM2 expressing lentiviral plasmids were obtained from the DNASU Plasmid Repository (Arizona State University, USA).

### Generation of XBP1 knockout clones

Parental MCF7 cells were electroporated with two gRNA plasmids (5’-ACTTTAGGGGTCCCGTCGGC-3’ and 5’-CCCGTCGGCCGGGTTCGGCG-3’) targeting XBP1 and Cas9 plasmid using ‘Nucleofector Kit V’ (Cat# VCA-1003) and 4D nucleofector (Lonza) following the manufacturer's instructions. Transfected cells were selected using puromycin (1 μg/ml), single cell clones were isolated, and loss of XBP1 was assessed by western blotting and PCR.

### Generation of RRM2 and CDC6 overexpressing clones

RRM2 overexpressing clones were generated using lentiviral transduction. Positive clones were selected using blasticidin containing media (5 µg/ml) for 1 week. CDC6 overexpressing clones were generated using retrovirus transduction and positive clones were selected by growing them in G418 containing media (800 µg/ml) for 2 weeks.

### RNA extraction, cDNA synthesis, and qPCR

The qRT-PCR used here has been described previously [13]. Briefly, Total RNA was isolated using Trizol (Invitrogen) and cDNA synthesis was carried out using ImProm-II™ Reverse Transcription System (Promega). Expression of genes of interest was carried out using predesigned prime time qPCR assays (Integrated DNA Technologies, Belgium) and StepOnePlus thermocycler (Applied Biosystems). Relative expression was calculated using the ΔΔCT method.

### Cell proliferation assay

MTS cell viability assay has been previously described [14]. Briefly, cells (2,000 cells/well) were plated in 96-well plate using DMEM media containing 10% FCS. After indicated days, MTS + PMS was added into each well and incubated at 37 °C for 4 h. The O.D was measured at 490 nm.

### Antibodies

Rabbit Beta-Actin antibody (Cat# A5060) was from Sigma Aldrich, Wicklow, Ireland. Mouse m-Ab XBP1s Ab (Cat# 647,502) was from Biolegend, London, UK. Mouse ER-α Ab (Cat# sc-8002), Mouse RRM2 Ab (sc-376973), mouse CDC6 Ab (Cat# sc-9964) were purchased from Santa Cruz Biotechnology, Inc. (Bergheimer, Heidelberg, Germany). Anti-Rabbit (Cat# 7074S) and anti-Mouse (Cat# 7076S) HRP linked secondary antibodies were purchased from cell signalling technology (Frankfurt, Germany).

### Western blot

The western blot used here has been described previously [15]. Briefly, protein samples were run on SDS-PAGE and transferred into the nitrocellulose membrane. Blocking was done using either 5% non-fat dry milk in phosphate-buffered saline (PBS)/0.05% tween-20 (for ERα, XBP1s, β-Actin, and CDC6) for 2 h or 2.5% milk + 2.5% bovine serum albumin in Tris-buffered saline (TBS)/0.1% tween-20 (for RRM2) for 2 h. After blocking, the membrane was incubated with primary antibody, ERα (1:1,000), XBP1s (1:1,000), CDC6 (1:500), Beta-Actin (1:2,000), and RRM2 (1:250) at 4 °C overnight. After washing the membranes were incubated with appropriate HRP-conjugated anti-rabbit (1:10,000) or anti-mouse (1:5,000) antibodies. Protein bands were visualized with Western Lightning® Plus-ECL, Enhanced Chemiluminescence Substrate (PerkinElmer, Llantrisant, UK).

### Cell death analysis

This has been described previously [16]. Cells (0.2 × 10^6^) were plated in a 6-well plate. After 24 h, cells were treated with either vehicle (0.1% DMSO) or with the required compounds for the indicated time points. The media was collected into a separate 15 ml tube, and cells were collected by trypsinization. The cells were then pelleted down by centrifugation at 1,200 rpm for 5 min at 4 °C, and media was discarded. Cells were then washed once with ice-cold PBS and resuspended in fluorescent activated cell sorting (FACS) buffer (100–150 µl) and transferred into a 1.5 ml Eppendorf tube. Before analysis, 4 µl (stock conc. 0.5 mg/ml) of propidium iodide (PI) was added to 100 µl of cell suspension and vortexed gently. Gating was done using unstained cells (without PI) and positive control cells were stained with PI. The percentages of dead cells (PI-positive cells) were determined by using BD Accuri C6 flow cytometer (BD Biosciences, Wokingham, UK).

### Cell cycle analysis

Cells (0.2 × 10^6^) were plated in a 6-well plate with DMEM medium. After 48 h of plating, cells were collected by trypsinization into a 15 ml tube. Cells were pelleted down by centrifugation at 1000 rpm, 4 °C for 5 min, media was discarded and cells were re-suspended in 1 ml of ice cold PBS. Cells were then washed twice with 1 ml of ice-cold PBS by centrifugation at 1000 rpm, for 5 min at 4 °C, PBS was discarded, and the cells were re-suspended in ice-cold 70% ethanol and kept overnight at -20 °C. The following day, cells were pelleted by centrifugation at 1000 rpm for 5 min at 4 °C, ethanol was discarded. Then cells were washed twice by centrifugation (1000 rpm for 5 min at 4 °C) with 1 ml of ice-cold PBS followed by re-suspension in 200 µl of RNase (20 μg/ml) containing PBS. Cells were transferred into a 1.5 ml Eppendorf tube. Then 5 µl of propidium iodide (PI, stock 0.5 mg/ml) was added into 200 µl of cell suspension and kept in a dark environment at least 30 min before analysis. Cell cycle analysis was carried out by using BD Accuri C6 flow cytometer (BD Biosciences, Wokingham, UK).

### Assessment of protein half-life

To determine the protein half-life, cells were plated in a 6-well plate and treated with cycloheximide (100 μg/ml) (Sigma Aldrich) for 0 h, 4 h, 8 h, and 16 h. After the indicated time point, whole cell lysates were prepared. Western blotting for whole cell lysates was performed to determine the expression of ER in control and XBP1 KO MCF7 cells.

### Cell synchronization using CSS

For all experiments with estradiol treatment, cells were always cultured in phenol red-free DMEM and charcoal-stripped serum (CSS). MCF7 WT, MCF7 XBP1 KO, MCF7 Y537S, and MCF7 D538G cells were synchronized by growing in phenol red-free DMEM containing 5% CSS for three days. For control and XBP1 KO MCF7 cells, synchronization and estradiol treatment experiments were carried out using phenol-red free DMEM containing 3% CSS. Cells were plated using phenol-red free DMEM containing 3% CSS and kept for synchronization. After synchronization, cells were treated with either vehicle or 17-estradiol for the indicated time points.

### Statistical analysis

The data was analysed using GraphPad Prism 5.01. Data is presented as mean ± SD from three independent experiments unless otherwise stated. Densitometry analysis was carried out using Image J software. The survival of breast cancer patients was determined using Kaplan–Meier analysis. Other analyses of datasets are indicated in the figure legend. *P*-value was determined using Student’s t-test between independent groups, results with *p* < 0.05 were considered statistically significant.

## Results

### Increased expression of XBP1 in ER-positive breast cancer

Analysis of XBP1 expression between normal and tumour samples from several human cancers using GEPIA (gene expression profiling interactive analysis) showed the higher levels of XBP1 in tumours as compared to normal tissue except in pancreatic adenocarcinoma (SF 1). Notably highest expression of XBP1 was observed in samples from breast invasive carcinoma (SF 1). Next, we assessed XBP1 expression in tumour tissues as compared to tumour adjacent and healthy tissues using GTEx and TCGA dataset (bc-GenExMiner v4.8). The analysis showed the higher expression of XBP1 in tumour tissues as compared to tumour adjacent and healthy tissues (SF 2). Further analysis of XBP1 expression in molecular subtypes of breast cancer showed highest expression in luminal subtype as compared to basal or HER2-enriched subtypes (SF 3A-B). Next, the expression of XBP1 mRNA was analysed in a panel of 50 breast cancer cell lines [luminal (*n* = 13), HER2-enriched (*n* = 16), basal-like (*n* = 21)] grouped according to molecular subtypes. In agreement with results in breast cancer tissue, cell lines also showed highest expression of XBP1 in luminal subtype (SF 3C). Next, we analysed the expression of XBP1 based on the ER status in the TCGA breast dataset (*n* = 593). The analysis showed a marked increase in expression of XBP1 mRNA in ER-positive breast cancer (SF 4). Taken together, these analyses indicate increased expression of XBP1 in ER-positive and/or luminal subtype of breast cancer.

### Generation of XBP1 deleted sub-clones of MCF7 cells

To understand the role of XBP1 in ER-positive breast cancer, we generated sub-clones of MCF7 cells with deletion of XBP1. MCF7 cells were co-transfected with XBP1-specific gRNA plasmids and a donor plasmid containing XBP1-homology arms and puromycin resistance gene. Firstly, a pool of cells were selected with puromycin (1 µg/ml) and evaluated by western blot. To induce the expression of XBP1s, MCF7 control and MCF7 XBP1 KO (pool) cells were treated with Brefeldin A (BFA). Robust induction of XBP1s was observed upon BFA treatment in MCF7 control cells. Western blot analysis confirmed the compromised induction of XBP1s in the pool of puromycin resistant cells. Subsequently, a total of 17 independent single cell clones were isolated from pool of puromycin resistant cells and evaluated for loss of XBP1s. Seventeen individual XBP1 KO MCF7 clones showed compromised expression of XBP1s to varying degrees with three independent sub-clones (clone# 9, clone# 15, and clone# 16) showing maximum effect on induction of XBP1s (SF 5). Clone# 9 and 16 were selected for subsequent analyses. Next, we checked the correct integration of donor plasmid (XBP1 homology arms with puromycin cassette) into the genome of XBP1 KO (#16) MCF7 cells. For this purpose, genomic DNA from MCF7 control cells and MCF7 XBP1 KO cells was isolated and three different sets of primers were used for genomic DNA PCR (SF 6A). As expected both 1st set and 2nd set of primers produced a PCR product of expected size when genomic DNA from the XBP1 KO (#16) MCF7 cells was used as a template and not from genomic DNA from control MCF7 cells (SF 6B). The 3rd set of primers was designed using the sequence from the outside of each homology arm and was predicted to produce a larger PCR product in MCF7 XBP1 knockout cells (3.6 kb) as compared to MCF7 control cells (1.25 kb). As expected, we observed a larger PCR product from genomic DNA of MCF7 XBP1 KO (#16) MCF7 cells as compared to control MCF7 cells (SF 6C). Together, these results confirm the correct integration of XBP1 homology arm and successful generation of XBP1 knockout sub-clones of MCF7 cells.

### Impaired induction of UPR target genes in XBP1 knockout MCF7 cells

**Fig. 1:**
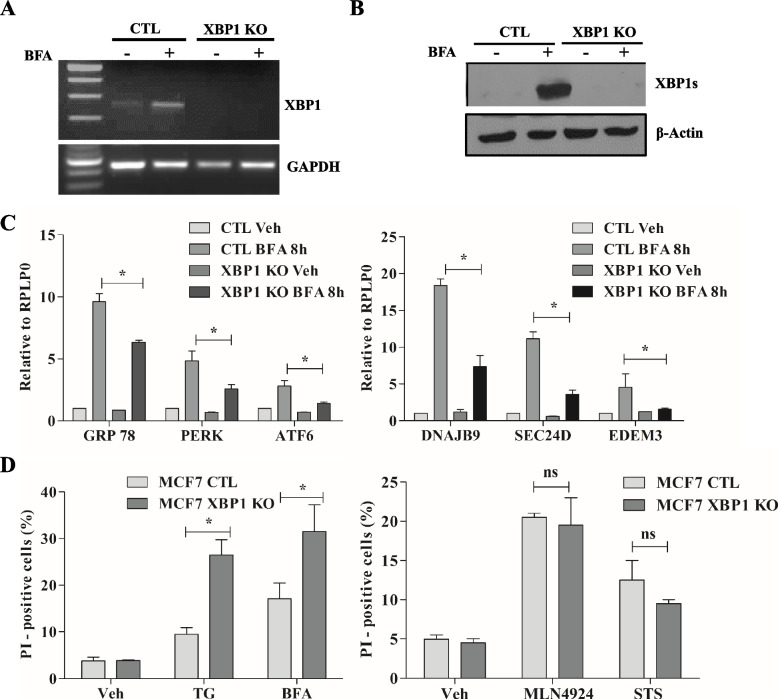
Compromised induction of UPR in XBP1 knockout MCF7 cells. **A-C** MCF7 control and MCF7 XBP1 KO (#16) cells were treated with BFA (2 µg/ml) for indicated time point. (A) Expression of XBP1 and GAPDH mRNA was determined by RT-PCR. **B** Western blotting was performed using antibodies against XBP1s and β-Actin. **C** Expression level of indicated genes was determined by qRT-PCR and normalised against RPLP0. Data presented is mean ± S.D (*n* = 3). **D** MCF7 control and MCF7 XBP1 KO #16 were treated with thapsigargin (TG), Brefeldin A (BFA), Pevonedistat (MLN4942) and staurosporine (STS) for 24 h. Propidium iodide (PI) positive cells are shown (*n* = 3). *p < 0.05, two-tailed unpaired t-test compared with control cells

MCF7 control cells and MCF7 XBP1 KO (#16) cells were treated with Brefeldin A (BFA) and induction of XBP1s mRNA and protein was determined. As shown in Fig. 1A-B, EnR stress mediated induction of XBP1s was absent in MCF7 XBP1 KO (#16) cells. We examined whether the absence of XBP1 affects the induction of UPR-target genes in response to EnR stress. The loss of XBP1 significantly attenuated the induction of UPR-target genes MCF7 cells (Fig. 1C). Almost the same extent of reduction was observed for the expression of UPR-target genes in another independent XBP1 KO sub-clone (#9). These results confirmed the role of XBP1s as a critical regulator of induction of UPR-target genes in response to EnR stress. Next we investigated the consequences of impaired induction of UPR-target genes in XBP1 KO (#16) MCF7 cells by evaluating cell death after treatment with EnR stress inducers. After 24 h of BFA and thapsigargin (TG) treatment, cell death was analysed using propidium iodide (PI) staining. The analysis showed a significantly increased EnR stress-induced cell death in XBP1 KO (#16) cells as compared to MCF7 control cells (Fig. 1D). Next, to determine whether XBP1-deficient MCF7 cells were specifically sensitive to UPR-induced cell death MCF7 XBP1 KO (#16) and MCF7 control cells were treated with MLN4924 and staurosporine. MLN4924 is a neddylation inhibitor that blocks neddylation of Cullins and triggers apoptosis in cancer cells independent of UPR. Staurosporine is an alkaloid that inhibits multiple kinases and highly potent inductor of UPR-independent apoptosis. Cell death analysis with MLN4924 and staurosporine treatment demonstrated no significant difference in cell death between MCF7 control cells and XBP1-deleted MCF7 cells (Fig. 1D). These results suggest that loss of XBP1 sensitizes MCF7 cells specifically to EnR stress-induced cell death.

### Deletion of XBP1 reduces cell growth and proliferation

**Fig. 2:**
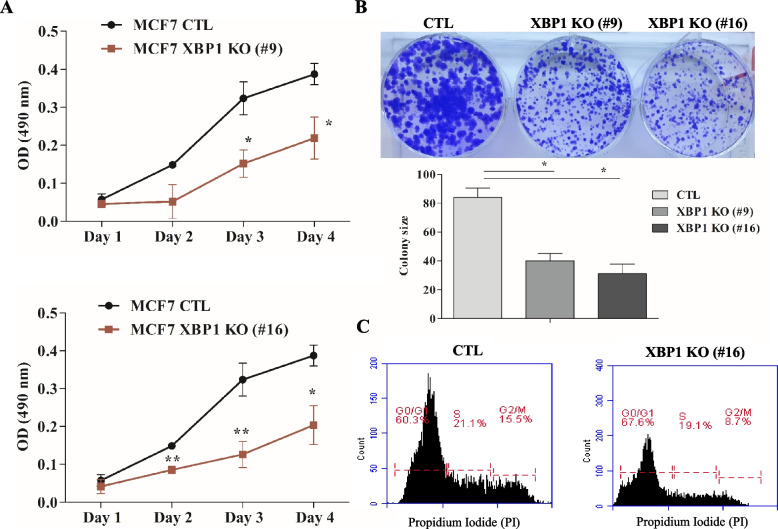
XBP1-deficient cells show reduced proliferation and accumulate in G1 of cell cycle. **A** MCF7 control and XBP1 knockout sub-clones of MCF7 cells (#9 and #16) were plated in 96-well plate. Line graphs show change in O.D at 490 nm. Data presented is mean ± SD from six replicates (*n* = 3) (**B**) MCF7 control and XBP1 knockout sub-clones were seeded in 6-well plate (1000 cells/well) and maintained for 14 days. Crystal violet stained colonies are shown. Lower panel shows the quantification of size and number of colonies per well. Data shown is mean ± S.D (*n* = 3). *p < 0.05, two tailed unpaired t-test compared with controls. **C** Cell cycle analysis of control and XBP1 KO (#16) MCF7 cells using PI staining followed by flow cytometry. Representative data of three independent analyses is shown

Next we performed cell viability and colony formation assays to evaluate the effect of XBP1 deletion on cell growth and proliferation in MCF7 cells. The MTS assay demonstrated a significant inhibition in cell growth of MCF7 XBP1 KO cells in comparison to MCF7 control cells (Fig. 2A). Approximately 50% reduction in cell viability was observed after 3 days of culture. The extent of growth inhibition was comparable in two independent XBP1 KO sub-clones (#9, #16) of MCF7 cells. Next, colony formation assay was performed using MCF7 control and XBP1 KO sub-clones of MCF7 cells (#9, #16). After 2 weeks of culture, staining with crystal violet demonstrated a notable reduction in colony formation in both XBP1 knockout sub-clones of MCF7 cells as compared to the MCF7 control cells (Fig. 2B). The quantification of colonies using Image J showed that colonies were two times smaller in size for XBP1-deleted MCF7 cells as compared to MCF7 control cells (Fig. 2B). To identify whether reduced cell proliferation in XBP1 KO MCF7 cells was associated with the altered cell cycle progression, cells were stained by PI and subjected to flow cytometry. The cell cycle analysis revealed that XBP1 KO (#16) cells had increased distribution of cells in G0/G1 phase but reduced distribution in G2/M-phase (Fig. 2C) as compared to control cells. Distribution of cells was almost the similar in S-phase in both MCF7 control cells and MCF7 XBP1 KO (#16) cells.

### XBP1 modulates optimal induction of estrogen-stimulated genes and response to anti-estrogens

**Fig. 3:**
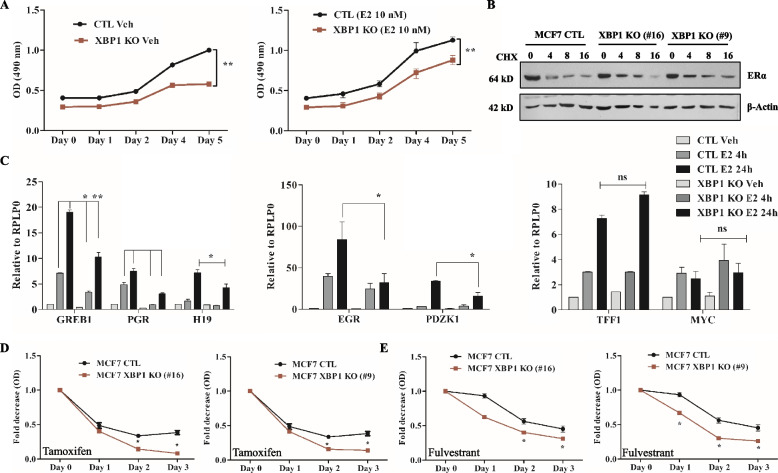
Loss of XBP1 attenuates induction of estrogen-responsive genes and sensitizes MCF7 cells to anti-estrogens. **A** After synchronization, cells were either treated with (Vehicle) DMSO or (10 nM E2) estrogen. Line graphs show change in O.D at 490 nm after E2 treatment. Data shown is mean ± S.D (*n* = 6). *p < 0.05, two tailed unpaired t-test. **B** Cells were treated with cycloheximide (CHX, 100 μg/ml) and expression of ERα and β-Actin was determined by western blotting. Representative western blot from three independent experiments is shown. **C** MCF7 control and XBP1 KO cells were treated with either vehicle (DMSO) or (10 nM) E2 for the required time points. Relative expression of indicated genes was analysed by qRT-PCR (*n* = 3), RPLP0 was used to normalize the gene expression. Data presented as mean ± SD. *p*-values were determined using two-tailed unpaired t-test, *p < 0.05, **p < 0.01, ns- not significant. (D) Cells were treated with (10 µM) tamoxifen and (10 µM) fulvestrant for indicated time points. Line graphs show change in O.D at 490 nm. Data presented is mean ± S.D of five independent experiments. *p < 0.05, two-tailed unpaired t-test comparing respective time points

We determined the effect of XBP1 deficiency on estrogen-dependent cell growth. MCF7 cells were synchronised by growing in phenol red-free DMEM and 3% CSS for 3 days and treated with (ranging from 1 pico mole – 100 nano mole) estrogen. Cultures were further incubated at 37 °C after which cell growth was monitored by MTS assay for up to 3 days after estrogen treatment. We found that 10 Nano moles of estrogen was optimal for continued growth of MCF7 cells up to 3 days after estrogen treatment (SF 7A). To evaluate estrogen-dependent cell growth, control and XBP1 KO cells were grown in phenol red free DMEM and 3% CSS for 3 days and cells were treated with 10 nM estrogen followed by MTS cell viability assay. Both vehicle and estrogen treated cells showed reduced cell growth in MCF7 XBP1 KO cells as compared to MCF7 control cells (Fig. 3A). Estrogen is the main stimulator for ER-positive breast cancer cell growth and its mitogenic effects are mediated by estrogen receptorα (ER). Therefore, we evaluated whether the deletion of XBP1 has any effect on the expression of the ER protein. MCF7 control and MCF7 XBP1 KO (#16, #9) cells were treated with cycloheximide (CHX) followed by immunoblotting for ER. We observed no difference in steady state expression as well as half-life of ER protein in XBP1 knockout MCF7 cells and MCF7 control cells (Fig. 3B). Therefore, the observed reduction in cell growth and proliferation of MCF7 XBP1 KO cells is not due to the altered ER protein expression.

Spliced XBP1 (XBP1s) physically interacts with ER and overexpression of XBP1s confers estrogen-independent cell proliferation and provides endocrine resistance [17]. Therefore, we hypothesized that the deletion of XBP1 could have effect on the expression of estrogen (E2)-responsive genes. Indeed, we observed a compromised induction of the ER-responsive genes in MCF7 XBP1 KO (#16) cells (Fig. 3C) as compared to control MCF7 cells. Activation of GREB1, PGR, H19, EGR, and PDZK1 genes was significantly compromised upon E2-stimulation in XBP1-deleted MCF7 cells as compared to the MCF7 control cells (Fig. 3C). The induction of GREB1, PGR, H19, EGR and PDZK1 was reduced by two-fold at after 24 h of E2 treatment. Noteworthy, the basal expression of some of the E2-stimulated genes, including GREB1, PGR, and EGR, was also reduced in XBP1-deleted MCF7 cells (Fig. 3C). However, the E2-induced expression of other ER-responsive genes, including MYC and TFF1 was not significantly different between control and XBP1 KO cells (Fig. 3C). Similar effect on expression of E2-responsive genes was observed in MCF7 XBP1 KO (#9) cells (SF 8). These results indicate that the deletion of XBP1 affects the basal and E2- stimulated expression of a sub-set of ER-target genes in MCF7 cells. Next, we determined effect of XBP1-deficiency on the sensitivity of MCF7 cells towards tamoxifen and fulvestrant. We found that 10 µM of tamoxifen and fulvestrant was optimal to study their growth inhibitory effects on MCF7 cells (SF 7B-C). We observed significantly enhanced sensitivity of XBP1-knockout MCF7 cells upon tamoxifen (Fig. 3D) and fulvestrant (Fig. 3E) treatment. Both the sub-clones of MCF7 (clones #9 and #16) lacking XBP1 exhibited comparable level of hypersensitivity towards anti-estrogens (Fig. 3D). These results confirm the role of XBP1 in regulating the cellular response towards anti-estrogens.

### Identification of XBP1-regulated genes in the context of ER-positive breast cancer

To identify the XBP1-regulated targets, we analysed transcriptomic and XBP1 ChIP-seq dataset from control and XBP1-targeting shRNA expressing T47D cells (GSE49955). A direct target gene was defined by its differential expression upon knockdown of XBP1 and occupancy of XBP1 at the gene locus. Based on the fold expression, novelty, and function, we short listed 13 different genes (Supplementary table 1) for validation. The qRT-PCR analysis in MCF7 control and MCF7 XBP1 KO (#16, #9) cells revealed downregulation of RRM2, CDC6, TOP2A, BIK, TNFSF10, and VTCN1 (SF 9A) in both XBP1 KO sub-clones. Next, we determined the expression of 13 short listed XBP1-target genes in T47D cells treated with STF083010, a chemical inhibitor of IRE1. This inhibitor blocks IRE1 endoribonuclease activity and thus impairs the production of spliced XBP1. To confirm the function of this chemical inhibitor, we treated T47D cells with EnR stressor, BFA in absence and presence of STF083010, and the splicing of XBP1 was determined. We observed inhibition of XBP1 splicing with STF083010 treatment (SF 9B) which indicated the proper functioning of this inhibitor. We observed the reduced expression of RRM2, RAB31, TOP2A, CDC6, CDC20B, BTG2, BIK, and TNFSF10 upon STF083010 treatment of T47D cells (SF 9C). Since RRM2, CDC6 and TOP2A expression consistently was reduced in both XBP1 KO sub-clones of MCF7 cells and upon chemical inhibition of XBP1 in T47D cells, these three genes were selected for further analysis.

**Fig. 4:**
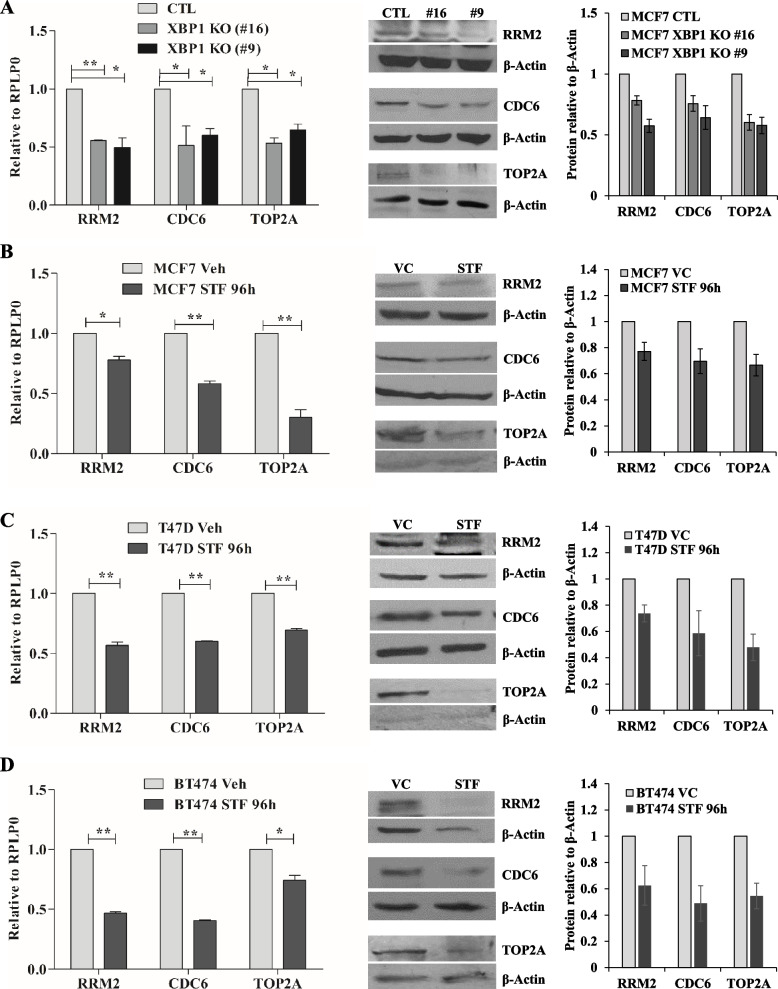
XBP1 is required for expression of RRM2, CDC6 and TOP2A. **A** Total RNA and whole cell lysate from control and XBP1 KO MCF7 cells was used to determine the expression of indicated genes. Expression level of indicated genes was evaluated by qRT-PCR and normalised against RPLP0. Data presented is mean ± S.D (*n* = 3). *p < 0.05, **p < 0.01, two-tailed unpaired t-test compared with control cells. Right panel, a representative immunoblot for RRM2, CDC6, TOP2 and β-Actin is shown (*n* = 3). **B-D** MCF7, T47D and BT474 cells were treated STF083010 (50 µM) for 96 h. Total RNA and whole cell lysate was used to determine the expression of indicated genes. Expression level of indicated genes was determined by real time RT-PCR and normalised against RPLP0. Data presented as mean ± S.D (*n* = 3). A representative immunoblot and quantification of RRM2, CDC6, TOP2A expression normalised to β-Actin is shown (*n* = 3)

We next determined the expression of RRM2, CDC6, and TOP2A mRNA and protein in XBP1-knockout sub-clones of MCF7 cells and ER-positive (MCF7, T47D, and BT474) breast cancer cells after treatment with STF083010. We found decreased expression of RRM2, CDC6, and TOP2A mRNA and protein upon genetic (Fig. 4A) and chemical (Fig. 4B-D) inhibition of XBP1 suggesting a role for XBP1 in regulating the expression of RRM2, CDC6, and TOP2A.

### Ligand independent expression of RRM2, CDC6 and TOP2A in ESR1 mutant MCF7 cells

**Fig. 5:**
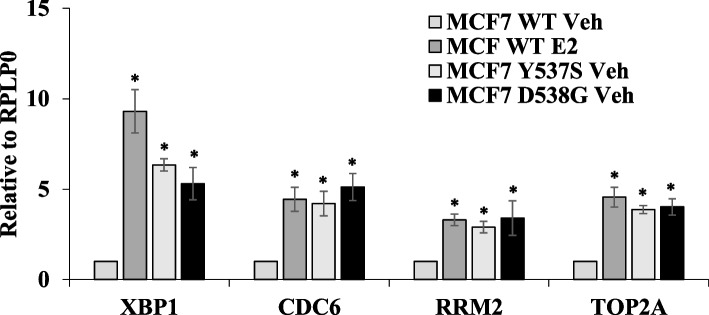
Ligand independent induction of RRM2, CDC6 and TOP2A in ESR1 mutant MCF7 cells. MCF7 WT, MCF7 Y537S, MCF7 D538G cells were synchronized for 3 days in phenol red free DMEM containing 5% CSS. MCF7 WT cells were either treated with (Veh) or estrogen (E2) for 24 h. Expression level of RRM2, CDC6, and TOP2A was evaluated by qRT-PCR and normalised against RPLP0. Data presented as mean ± S.D of three independent experiments. *p < 0.05, two-tailed unpaired t-test compared with vehicle treated MCF7 WT cells

Our results (Fig. 3C) showed that the deletion of XBP1 affects the basal and E2-stimulated upregulation of a sub-set of ER-target genes in MCF7 cells. Therefore, we evaluated estrogen-induced expression of RRM2, CDC6, and TOP2A. We used wild-type MCF7 cells and genome-edited MCF7 cells consisting Y537S and D538G mutation [18]. Somatic mutations in the ligand-binding domain of ESR1 have been found in up to 40% of metastatic, endocrine-resistant ER-positive breast cancer patients. Two most prevalent ESR1 missense mutations (Y537S and D538G) show constitutive, estrogen-independent transcriptional activity, and partial-resistance to hormonal therapy. Wild-type MCF7, MCF7-Y537S and MCF7-D538G cells were grown in steroid free medium and the expression of RRM2, CDC6, and TOP2A including XBP1 was analysed. We observed an increased expression of all three genes upon estrogen stimulation of wild-type MCF7 cells (Fig. 5). Interestingly, in genome-edited cells (MCF7-Y537S and MCF7-D538G cells), the extent of RRM2, CDC6, and TOP2A gene expression in steroid free conditions was comparable to estrogen-induced expression of wild-type MCF7 cells (Fig. 5). Indeed, analysis of promoter regions of RRM2, CDC6 and TOP2A by CiiiDER, algorithm for predicting and analysing putative TFBSs within regulatory regions identified binding sites for both ESR1 and XBP1 (SF 10). These results suggest that expression of RRM2, CDC6 and TOP2A is responsive to both XBP1 and ER.

### Expression of RRM2 and CDC6 rescues hypersensitivity of XBP1 KO cells

**Fig. 6:**
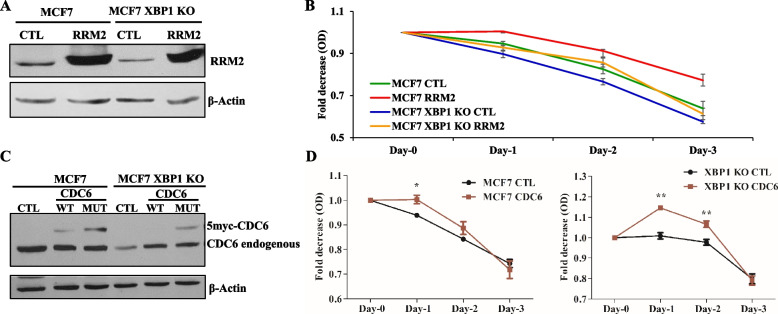
Expression of RRM2 and CDC6 rescues tamoxifen hypersensitivity in XBP1 knockout MCF7 cells. **A** Whole cell lysates from indicated RRM2 expressing cells were analysed by western blotting using antibodies against RRM2 and β-Actin. **B** Indicated RRM2 expressing cells were treated with tamoxifen (10 µM) for required time points. Line graphs show change in O.D at 490 nm. Data presented is mean ± S.D (*n* = 4). *p < 0.05, two-tailed unpaired t-test comparing respective time points. **C** Whole cell lysates from indicated (wild type and mutant) CDC6 expressing cells were subjected to western blotting using antibodies against CDC6 and β-Actin. **D** Indicated mutant CDC6 expressing cells were treated with tamoxifen (10 µM) for required time points. Line graphs show the change in O.D at 490 nm. Data presented as mean ± SD (*n* = 3). *p < 0.05, **p < 0.01 from two-tailed unpaired t-test comparing respective time points

Next, we tested whether reduced expression of RRM2 or CDC6 genes is responsible for the observed tamoxifen hypersensitivity of the XBP1-deleted MCF7 cells. For this, we generated RRM2 expressing clones in MCF7 and MCF7 XBP1 KO (#16) cells (Fig. 6A). The restoration of RRM2 expression partially rescued the reduced growth of XBP1-deleted MCF7 cells. Next, RRM2 expressing MCF7 control and MCF7 XBP1 KO (#16) cells were treated with tamoxifen (10 µM) for indicated days and the cell viability was determined using MTS assay. We observed that ectopic expression of RRM2 partially reversed the increased sensitivity of MCF7 XBP1 KO cells towards tamoxifen (Fig. 6B). Thus, restoration of RRM2 expression in XBP1-deleted MCF7 cells can partially rescue the phenotypes (reduced growth and sensitivity to tamoxifen) of MCF7 XBP1 KO cells.

Next, we generated CDC6 expressing clones in MCF7 and MCF7 XBP1 KO (#16) cells. Wild-type CDC6 is an unstable protein and we could not detect the expression of ectopic wild-type CDC6 in XBP1 KO MCF7 cells, therefore we expressed mutant-CDC6 (R56A, L59A, K81A, E82A, and N83A) which is more stable and has longer half-life [19]. We observed the expression of both wild-type and mutant-CDC6 in control MCF7 cells with expression of mutant-CDC6 higher than wild-type CDC6 protein (Fig. 6C). In XBP1 KO MCF7 cells, we were able to detect the expression of mutant CDC6 (Fig. 6C). Next, mutant CDC6 expressing MCF7 control and MCF7 XBP1 KO cells were treated with tamoxifen (10 µM) for indicated days. The MTS assay demonstrated that ectopic expression of mutant-CDC6 in XBP1 KO MCF7 cells partially reversed the sensitivity towards tamoxifen whereas, in control MCF7 cells mutant CDC6 showed a slight resistance towards tamoxifen treatment (Fig. 6D). Together, these data suggest a role for RRM2 and CDC6 in mediating endocrine resistance downstream of XBP1 in MCF7 cells.

### Prognostic value of XBP1-gene signature in ER-positive breast cancer

**Fig. 7:**
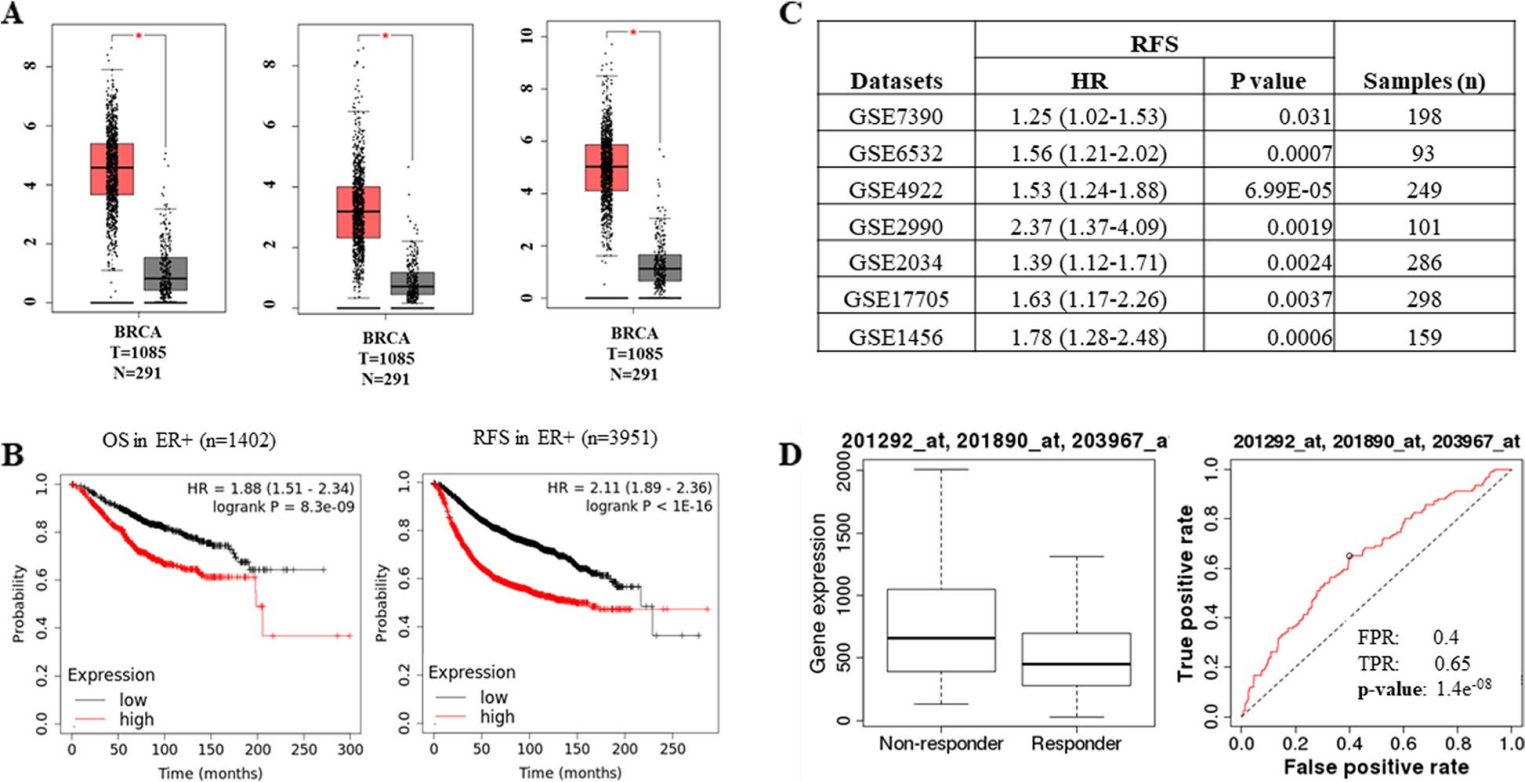
Association of XBP1-gene signature with outcome in ER-positive breast cancer. **A** Box plot for expression of RRM2, CDC6 and TOP2A in tumour and normal tissues in human breast cancers is shown. Median is shown by horizontal black line, the box is the upper and lower quartiles and the two lines outside the box show the highest and lowest values. **B** KM Plotter (https://kmplot.com/analysis/) was used to determine the association of XBP1-gene signature (CDC6, RRM2 and TOP2A) with overall survival (OS) and recurrence free survival (RFS) in ER-positive breast cancer. **C** Web-based algorithm PROGgeneV_2_ (http://www.progtools.net/gene/index.php) was used to test the association between XBP1-gene signature and RFS in indicated datasets of breast cancer. **D** Web-based algorithm ROC plot (http://www.rocplot.org/) was used to evaluate the association between XBP1-gene signature and response to tamoxifen treatment in ER-positive breast cancer

The analysis of the expression of RRM2, CDC6, and TOP2A in normal and tumour breast invasive carcinoma using GEPIA showed significant increased expression of RRM2, CDC6 and TOP2A in tumour samples as compared to normal tissue (Fig. 7A). We determined association of XBP1-gene signature (RRM2, CDC6 and TOP2A) with the outcome in breast cancer. Survival analysis using breast cancer data sets by KM plotter revealed that increased expression of XBP1-gene signature was strongly associated with shorter overall survival (OS) (hazard ratio: 2.22, 95% confidence interval: 1.7–2.9, *p* < 8.3E-9) and relapse free survival (RFS) (hazard ratio: 2.17, 95% confidence interval: 1.9–2.48, *p* < 1E-16) in ER-positive breast cancer (Fig. 7B). We did not find any significant association between the expression of XBP1-gene signature and outcome in either basal or HER2 + subtypes of breast cancer (SF 11). Further, XBP1-gene signature was associated with poor RFS among different cohorts of breast cancer patients (Fig. 7C). We next analysed the expression of RRM2, CDC6 and TOP2A in two breast cancer patient datasets pre- and post-endocrine treatment (GSE10281, GSE80077). We observed a significant decrease in the expression of RRM2, CDC6 and TOP2A along with few bonafide estrogen-responsive genes (GREB1 and PDZK1) upon treatment with endocrine therapy, most likely due to inhibition of ER activity in response to endocrine therapy (SF 12). This is in agreement with our results showing increase in expression of RRM2, CDC6 and TOP2A upon estrogen stimulation of wild-type MCF7 cells (Fig. 5). Next, we evaluated the utility of XBP1-gene signature as predictive biomarker of response to tamoxifen (http://www.rocplot.org). We found higher expression of XBP1-gene signature in the non-responders and ROC analyses according to 5-year Relapse-Free Survival (RFS) upon tamoxifen treatment yielded AUC of 0.647 (Fig. 7D). These results show that increased expression of XBP1-gene signature (RRM2, CDC6 and TOP2A) in ER-positive breast cancer is associated with shorter survival and resistance to tamoxifen in ER-positive breast cancer.

## Discussion

Multi-omics analysis of primary breast tumour samples of TCGA cohort has shown that one of the salient feature of ER-positive breast cancers is increased expression of ER and XBP1 at mRNA and protein level [20]. Using web-based analysis of several breast cancer cohorts, we confirmed higher expression of XBP1 in ER-positive/luminal breast cancer. Extranuclear, non-genomic signalling upon estrogen stimulation activates the anticipatory UPR by causing a release of calcium from EnR to the cytoplasm [21]. Upon estrogen stimulation, ER increases the expression of XBP1 by binding to the enhancer region of XBP1 gene [22]. MYC, an estrogen-responsive gene upregulates the expression of IRE1 during estrogen stimulation [23]. Thus, estrogen signalling increases the expression of both IRE1 and XBP1 leading to sustained activation of IRE1-XBP1 axis, which augments the production of XBP1s. Indeed, expression of the XBP1s protein is elevated by estrogen stimulation in ER-positive breast cancer cells [22, 24, 25]. Further, XBP1s protein physically interacts with ER and enhances the estrogen-independent transactivational function of ER [12, 26] which generates a positive feedback loop consisting of XBP1s and ER. This positive feedback loop generated by cross talk between estrogen signalling and UPR contributes to elevated co-expression of ER and XBP1 in ER-positive breast cancer [3].

The IRE1-XBP1 axis is an evolutionarily conserved branch of the UPR pathway that plays a major role in cellular adaptation during EnR stress [27]. Increased expression of XBP1-target genes that augments EnR protein handling capacity and clearance of misfolded and/or unfolded proteins plays a crucial role in mediating pro-survival function of XBP1 [7, 28]. It has been reported that level of spliced XBP1 mRNA decline during prolonged EnR stress, whereas activity of PERK pathway is maintained over time [29, 30]. Reduction of XBP1 splicing was shown to coincide with induction of cell death during irreversible EnR stress and experimental potentiation of IRE1-dependent XBP1 splicing protected cells from EnR stress-induced cell death [29, 30]. Furthermore, XBP1s is essential for the survival of several secretory cell types and reduced XBP1 expression compromises cell survival under conditions of chronic EnR stress [31]. In keeping with these observations, we show that loss of XBP1 impaired induction of UPR-target genes and sensitized MCF7 cells specifically to EnR stress-induced cell death.

Loss of XBP1 has been reported to increase the sensitivity to hypoxia-mediated death of transformed cells and markedly suppress tumour growth in mouse embryonic fibroblasts and human fibrosarcoma cells [32]. Expression of XBP1s is increased in endocrine-resistant breast cancers, and ectopic XBP1s confers estrogen-independent cell growth and resistance to anti-estrogen therapy [3]. Gene expression profile induced during transient, non-lethal EnR stress induced by HyT36-mediated disruption of the ERHT protein folding showed induction of estrogen-stimulated genes [33]. Furthermore, inhibition of IRE1-XBP1 signalling impaired the upregulation of the estrogen-responsive genes by misfolded ERHT suggesting that ER activity induced by misfolded ERHT is dependent on IRE1-XBP1 axis [33]. Engineered cells expressing the Y537S point mutant of ER show elevated flux through UPR signalling and increased basal expression of XBP1s, which was associated with endocrine resistance. Indeed, increased expression of XBP1s is associated with poorer clinical outcomes in ER-positive breast cancers treated with anti-estrogen therapy [34]. Further inhibition of XBP1 splicing using the chemical inhibitor STF083010 could reverse the resistance towards tamoxifen in MCF7 cells [35]. Hu et al., reported that XBP1 inhibition sensitizes the endocrine resistant breast cancer cells towards anti-estrogens [11]. In agreement with these findings [11], we also report reduced cell growth and proliferation of XBP1-deficient MCF7 cells and increased sensitivity towards anti-estrogens. Our results indicate that the deletion of XBP1 affects the basal and E2-stimulated expression of a sub-set of ER-target genes. Further work is required to reveal the molecular mechanisms behind the differential requirement of XBP1 for optimal induction of estrogen-responsive genes.

**Fig. 8:**
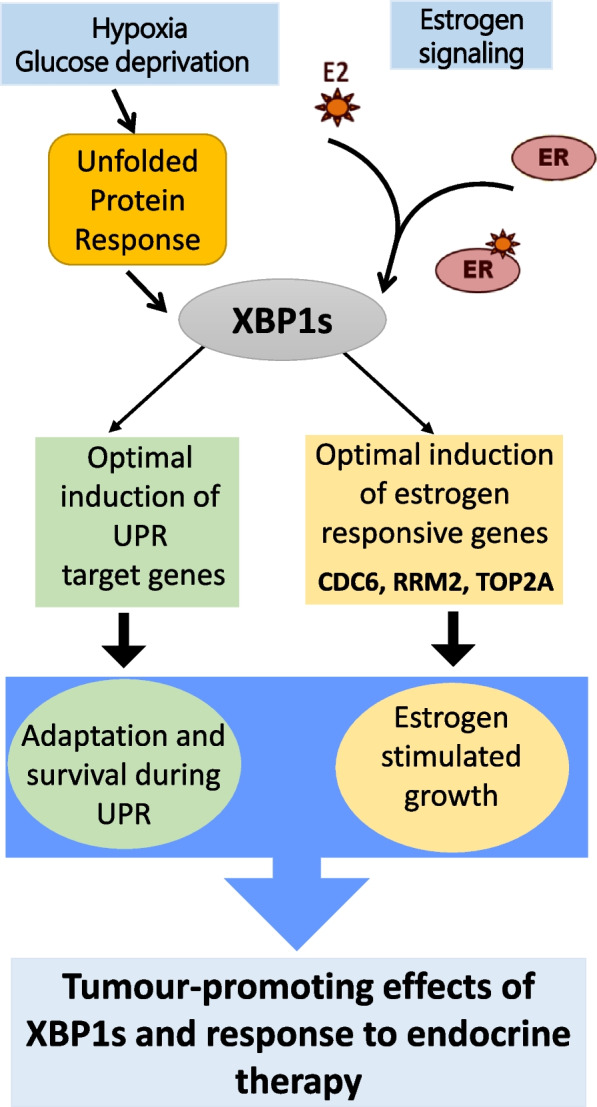
Graphical summary. Hypoxia and glucose deprivation are physiologically important inducers of unfolded protein response in tumour microenvironment. In estrogen receptor (ER) positive breast cancer cells XBP1s is induced in response to E2-stimulation and conditions of UPR. Tumour cells survive stressful conditions of microenvironment by an adaptive mechanism called the unfolded protein response (UPR). XBP1s is a transcriptional activator that regulates expression of genes involved in protein homeostasis and promote cell survival during conditions of UPR. XBP1s is required for optimal induction of E2-responsive genes and RRM2, CDC6 and TOP2A. RRM2 and CDC6 mediate endocrine resistance downstream of XBP1s in ER-positive breast cancer. ER, Estrogen receptor alpha; XBP1, X-box binding protein 1

Gene profiling studies to identify transcriptional network of XBP1 have shown that XBP1 primarily upregulates the genes involved in maintenance EnR homeostasis [8]. While the expression of XBP1 target genes associated with maintenance of EnR homeostasis is consistently observed among many tissues, there are few unique XBP1-target genes whose expression is restricted to the stimulus and cell type [8, 36]. Our results suggest that XBP1s regulates the expression of RRM2, CDC6 and TOP2A in ER-positive breast cancer (Fig. 8). RRM2, CDC6 and TOP2A have been previously reported as targets of XBP1s in other cell types (such as myocytes, plasma cells and pancreatic β-cells) [8] and helper T-cells [36]. Further, expression of RRM2, CDC6, and TOP2A was regulated by estrogen stimulation in ER-positive breast cancer. Indeed, several studies have reported estrogen-dependent expression of RRM2 [37, 38], CDC6 [39, 40], and TOP2A [38]. Further analysis of cistromic (ChIP-Seq) datasets at ‘The Signaling Pathways Project’ [41] revealed that both ER and XBP1 can to be recruited to the proximal promoter region of RRM2, CDC6, and TOP2A. Our results suggest that expression of RRM2, CDC6 and TOP2A can be regulated by concerted effort of both ER and XBP1 (Fig. 8). Analysis of identified XBP1-target genes (RRM2, CDC6 and TOP2A) using Cyclebase 3.0 [42], (Database on cell-cycle regulation and phenotypes) revealed their cell cycle dependent expression and key function in regulating the progression through different phases of cell cycle. We propose that reduced cell growth and altered cell cycle distribution of XBP1 KO cells is mediated in part by reduced expression of RRM2, CDC6 and TOP2A.

Our results demonstrate a role of RRM2 and CDC6 in mediating endocrine resistance downstream of XBP1 (Fig. 8). RRM2 catalyses a critical step in the DNA synthesis and repair pathway, and plays an important role in key biological processes such as cell growth, migration, invasion and senescence [43]. RRM2 was reported as a key mediator of AKT-induced resistance to tamoxifen and chemical inhibition of RRM2 by Didox reversed tamoxifen-resistant phenotype such as cancer growth, migration and invasion [44, 45]. Integrative metabolomics and gene expression revealed association of RRM2 expression with aggressive breast cancer and tamoxifen resistance, notably pharmacological or genetic inhibition of RRM2 sensitized tumours to tamoxifen treatment [46]. CDC6 plays a crucial role in the initiation of DNA replication in eukaryotic cells and exhibits oncogenic properties when overexpressed [47]. Expression of CDC6 is increased in bladder cancer and its knockdown can attenuate cell migration and invasion in addition to increased sensitivity to cisplatin [48]. CDC6 expression is upregulated in prostate cancer and its overexpression confers resistance to enzalutamide and the Chk1/2 inhibitor (AZD7762) [49]. Increased CDC6 expression is associated with poor survival and resistance to letrozole in the breast cancer patients [40]. We report an association between XBP1 gene signature (RRM2, CDC6 and TOP2A) and outcome as well as response to tamoxifen in ER-positive breast cancer. The efficacy of ORIN1001 (IRE1 inhibitor that blocks XBP1s production) is being evaluated in phase 1 trial in patients with advanced solid tumours or relapsed refractory metastatic breast cancer (NCT03950570). We propose that XBP1-gene signature may help in stratification of patients and serve as biomarkers of response to ORIN001.

## Conclusion

We show that XBP1 is required for optimal cell growth, upregulation of estrogen-responsive genes, and response to anti-estrogens. Expression of RRM2, CDC6 and TOP2A in ER-positive breast cancers is regulated by concerted efforts of both XBP1 and ER. RRM2 and CDC6 are mediators of endocrine resistance downstream of XBP1s. Finally, we show an association of XBP1-gene signature with poor outcome and response to tamoxifen in ER-positive breast cancer.

## Supplementary Information

**Additional file 1: SF1.** Expression of XBP1 in tumour and normal samples from different human cancers. **SF2.** Higher expression of XBP1 in breast tumour tissue. **SF3.** XBP1 expression in different molecular subtypes of breast cancer. **SF4.** Expression of XBP1 mRNA in TCGA breast dataset grouped according to ER status. **SF5.** Screening of XBP1 Knockout single cell clones in MCF7 cells. **SF6.** Confirmation of correct integration of XBP1 homology arm in XBP1 KO cells. **SF7.** Determination of optimal dose of estrogen, tamoxifen and fulvestrant for MCF7 cells. **SF8.** Loss of XBP1 attenuates induction of estrogen-responsive genes. **Supplementary Table 1.** XBP1-target genes shortlisted after the analysis of Gene Expression Omnibus dataset (GSE49955). **SF9.** Validation of XBP1 regulated genes. **SF 10.** ESR1 and XBP1-binding sites in the proximal promoter region of RRM2, CDC6, and TOP2A. **SF11.** Association between XBP1-gene signature and outcome in Basal and HER2-enriched breast cancer. **SF12.** Expression of RRM2, CDC6 and TOP2A in two breast cancer patient datasets pre- and post-endocrine treatment.

**Additional file 2.**

## Data Availability

All data used in this study was generated by experimental studies and available in this paper. Additional data referred in this article are attached as supplementary data. The dataset used in this study (GSE49955) can be found in the GEO database (https://www.ncbi.nlm.nih.gov/geo/query/acc.cgi?acc=GSE49955).

## Abbreviations

ATF6: Activating transcription factor 6α
BFA: Brefeldin A
CSS: Charcoal stripped serum
DMEM: Dulbecco’s Modified Eagle Medium
DMSO: Dimethyl sulfoxide
E2: Estrogen/β-estradiol
ECACC: European Collection of Authenticated Cell Cultures
EnR: Endoplasmic reticulum
ER: Estrogen receptor-α
FACS: Fluorescence-activated cell sorting
FCS: Fetal Calf Serum
IRE1: Inositol requiring enzyme 1α
KO: Knockout
OS: Overall survival
PERK: Double-stranded RNA-activated protein kinase-like ER kinases
PI: Propidium Iodide
RFS: Relapse free survival
SF: Supplementary Figure
TCGA: The Cancer Genome Atlas
TG: Thapsigargin
TM: Tunicamycin
UPR: Unfolded protein response
XBP1: X-box binding protein-1
NCOA3: Nuclear Receptor Coactivator 3
