# Supplementary material for: RRM2 and CDC6 are novel effectors of XBP1-mediated endocrine resistance and predictive markers of tamoxifen sensitivity
Source: BMC Cancer. 2023 Mar 30;23:288. doi: 10.1186/s12885-023-10745-1 (PMC10061897; doi:10.1186/s12885-023-10745-1)
Supplement: Supplementary file 1 — Additional file 1: SF1. Expression of XBP1 in tumour and normal samples from different human cancers. SF2. Higher expression of XBP1 in breast tumour tissue. SF3. XBP1 expression in different molecular subtypes of breast cancer. SF4. Expression of XBP1 mRNA in TCGA breast dataset grouped according to ER status. SF5. Screening of XBP1 Knockout single cell clones in MCF7 cells. SF6. Confirmation of correct integration of XBP1 homology arm in XBP1 KO cells. SF7. Determination of optimal dose of estrogen, tamoxifen and fulvestrant for MCF7 cells. SF8. Loss of XBP1 attenuates induction of estrogen-responsive genes. Supplementary Table 1. XBP1-target genes shortlisted after the analysis of Gene Expression Omnibus dataset (GSE49955). SF9. Validation of XBP1 regulated genes. SF 10. ESR1 and XBP1-binding sites in the proximal promoter region of RRM2, CDC6, and TOP2A. SF11. Association between XBP1-gene signature and outcome in Basal and HER2-enriched breast cancer. SF12. Expression of RRM2, CDC6 and TOP2A in two breast cancer patient datasets pre- and post-endocrine treatment. [file 12885_2023_10745_MOESM1_ESM.pptx]

## Slide 1
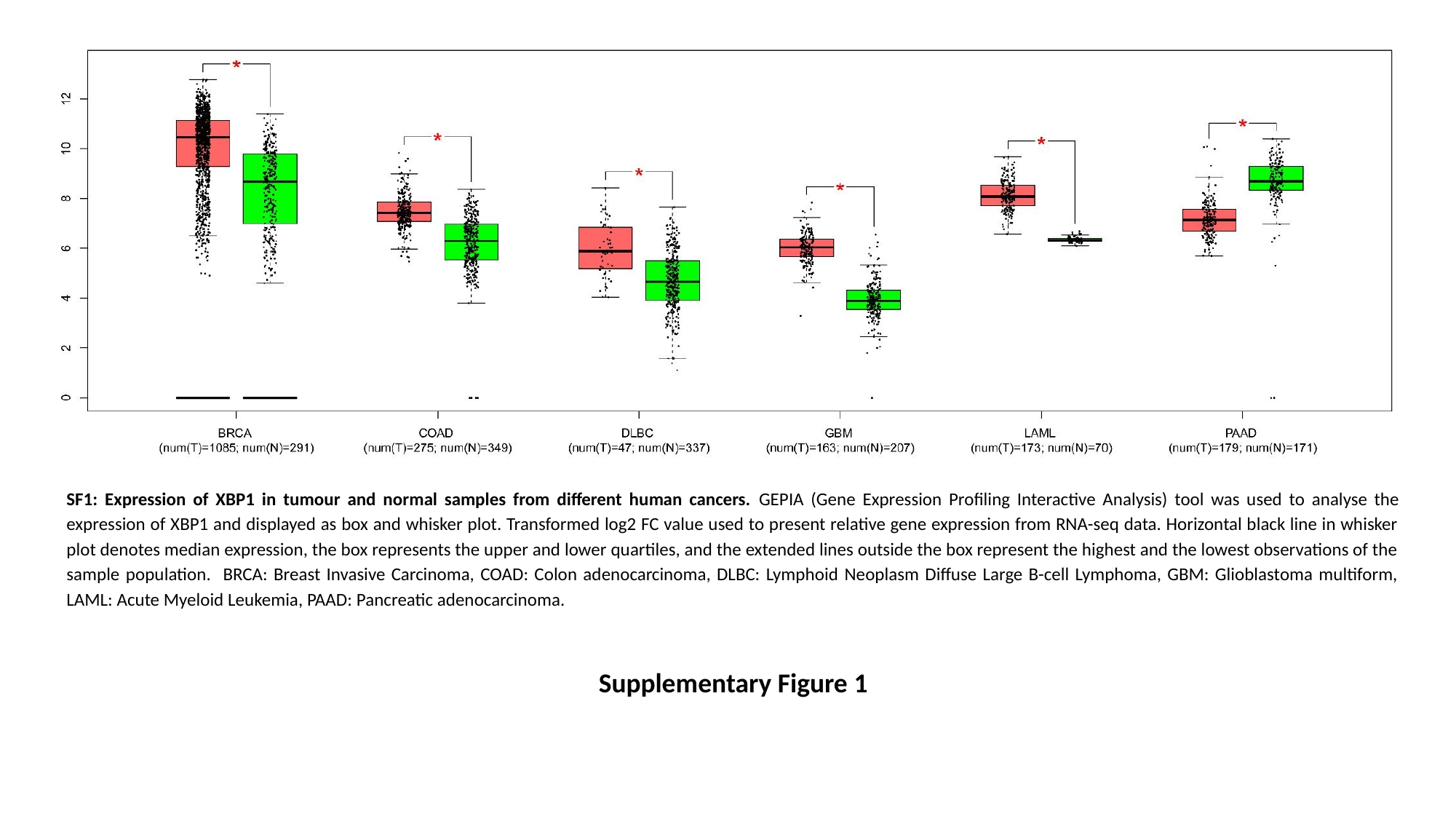

SF1: Expression of XBP1 in tumour and normal samples from different human cancers. GEPIA (Gene Expression Profiling Interactive Analysis) tool was used to analyse the expression of XBP1 and displayed as box and whisker plot. Transformed log2 FC value used to present relative gene expression from RNA-seq data. Horizontal black line in whisker plot denotes median expression, the box represents the upper and lower quartiles, and the extended lines outside the box represent the highest and the lowest observations of the sample population. BRCA: Breast Invasive Carcinoma, COAD: Colon adenocarcinoma, DLBC: Lymphoid Neoplasm Diffuse Large B-cell Lymphoma, GBM: Glioblastoma multiform, LAML: Acute Myeloid Leukemia, PAAD: Pancreatic adenocarcinoma.
Supplementary Figure 1

## Slide 2
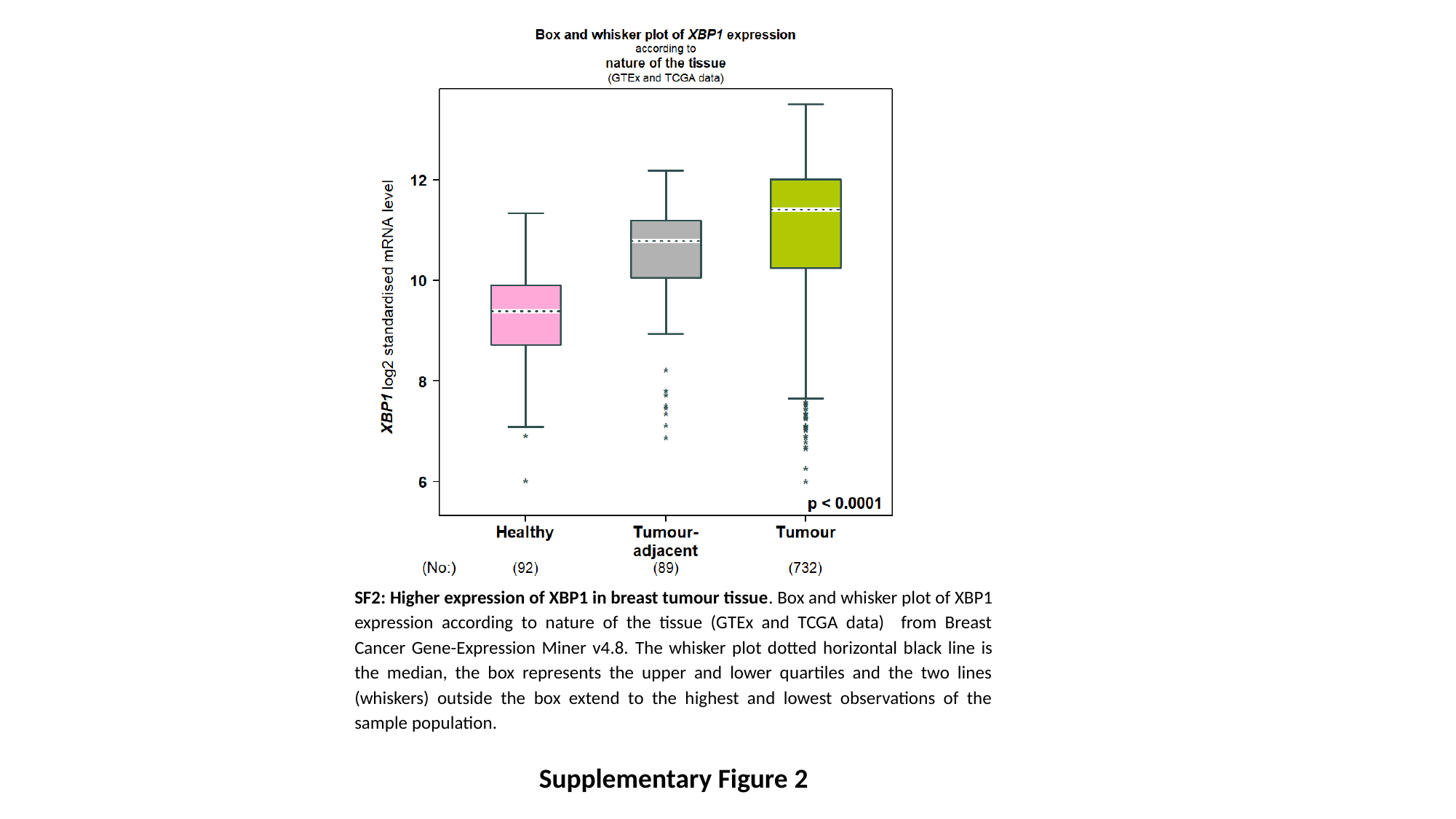

SF2: Higher expression of XBP1 in breast tumour tissue. Box and whisker plot of XBP1 expression according to nature of the tissue (GTEx and TCGA data) from Breast Cancer Gene-Expression Miner v4.8. The whisker plot dotted horizontal black line is the median, the box represents the upper and lower quartiles and the two lines (whiskers) outside the box extend to the highest and lowest observations of the sample population.
Supplementary Figure 2

## Slide 3
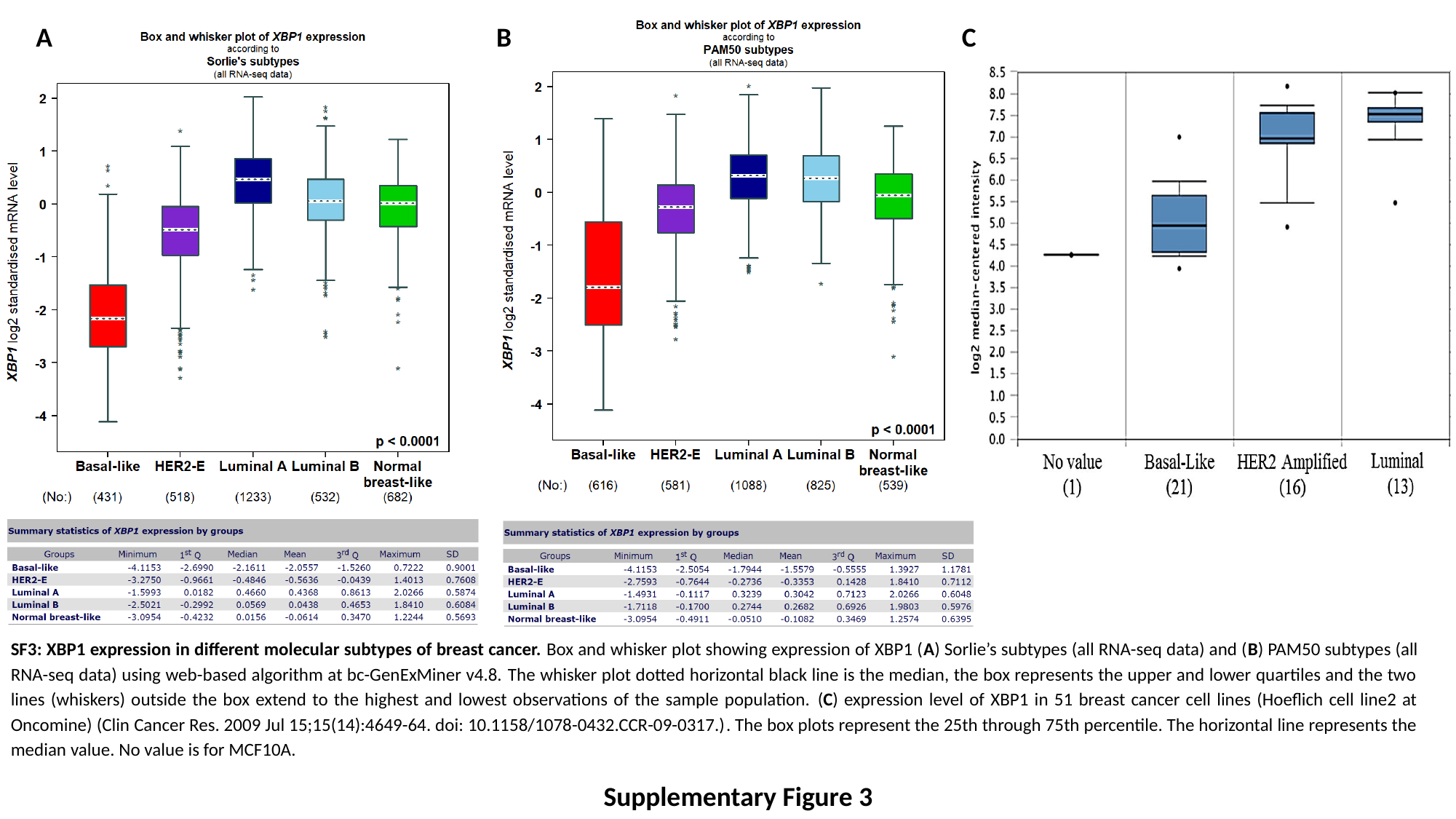

A B C
SF3: XBP1 expression in different molecular subtypes of breast cancer. Box and whisker plot showing expression of XBP1 (A) Sorlie’s subtypes (all RNA-seq data) and (B) PAM50 subtypes (all RNA-seq data) using web-based algorithm at bc-GenExMiner v4.8. The whisker plot dotted horizontal black line is the median, the box represents the upper and lower quartiles and the two lines (whiskers) outside the box extend to the highest and lowest observations of the sample population. (C) expression level of XBP1 in 51 breast cancer cell lines (Hoeflich cell line2 at Oncomine) (Clin Cancer Res. 2009 Jul 15;15(14):4649-64. doi: 10.1158/1078-0432.CCR-09-0317.). The box plots represent the 25th through 75th percentile. The horizontal line represents the median value. No value is for MCF10A.
Supplementary Figure 3

## Slide 4
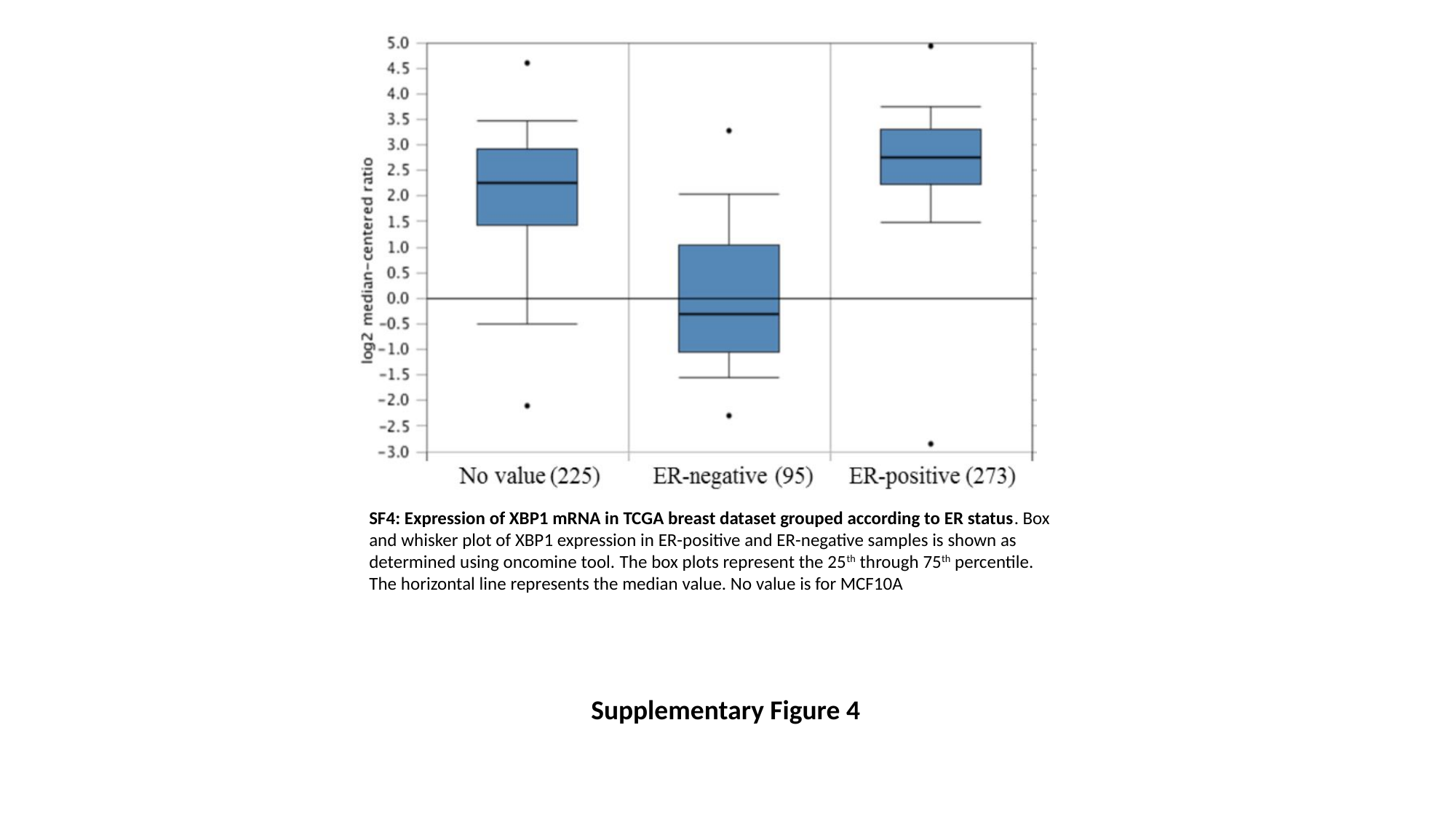

SF4: Expression of XBP1 mRNA in TCGA breast dataset grouped according to ER status. Box and whisker plot of XBP1 expression in ER-positive and ER-negative samples is shown as determined using oncomine tool. The box plots represent the 25th through 75th percentile. The horizontal line represents the median value. No value is for MCF10A
Supplementary Figure 4

## Slide 5
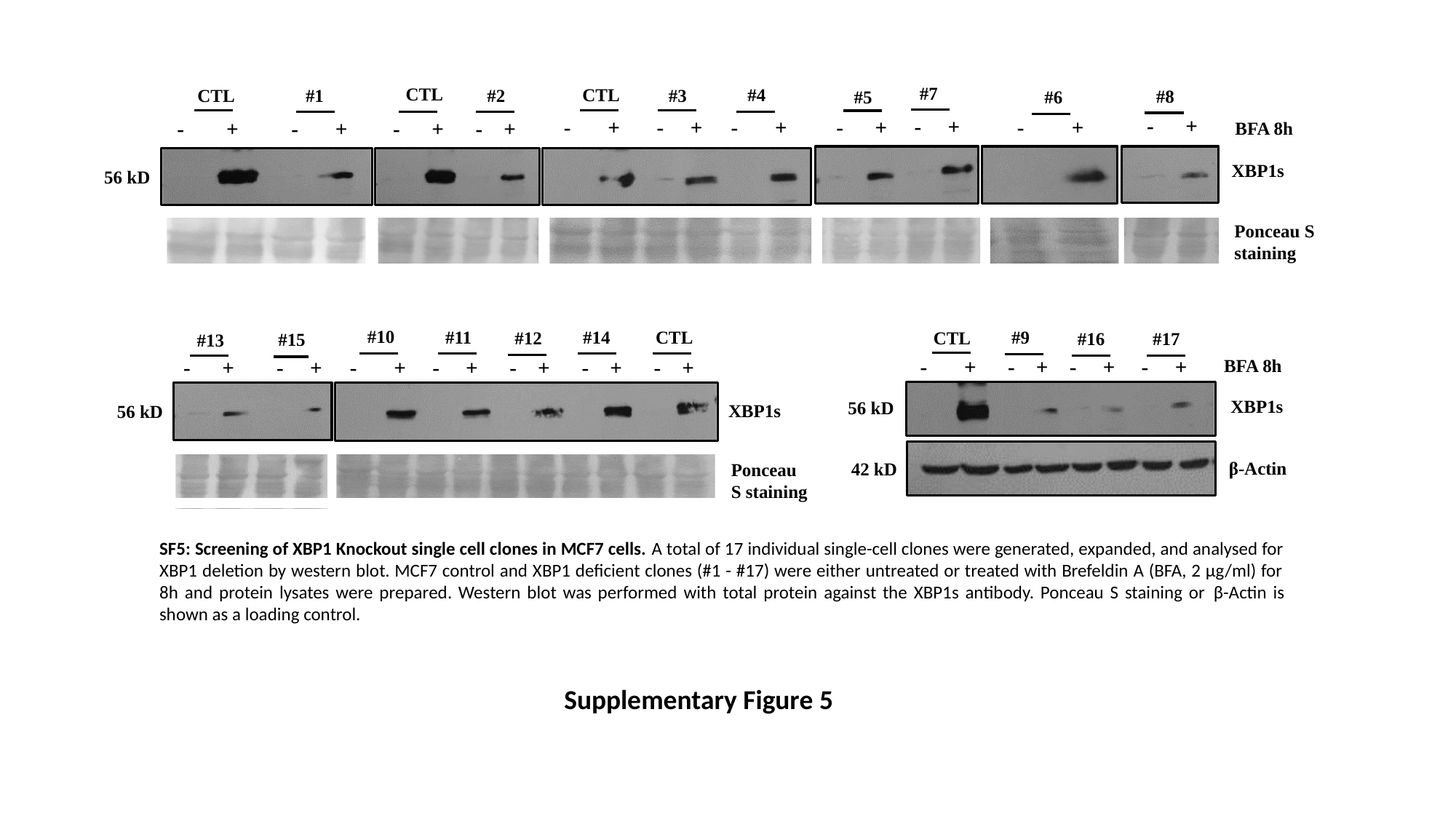

#7
CTL
#4
CTL
CTL
#1
#2
#3
#8
#5
#6
 - +
 - +
 - +
 - + - +
 - +
 - +
 - + - +
 - + - +
BFA 8h
XBP1s
56 kD
Ponceau S staining
#10
#14
#11
CTL
#12
#15
#13
 - + - + - + - + - +
 - + - +
56 kD
Ponceau S staining
#9
CTL
#17
#16
 - + - + - + - +
BFA 8h
XBP1s
XBP1s
56 kD
β-Actin
42 kD
β-Actin
XBP1s
SF5: Screening of XBP1 Knockout single cell clones in MCF7 cells. A total of 17 individual single-cell clones were generated, expanded, and analysed for XBP1 deletion by western blot. MCF7 control and XBP1 deficient clones (#1 - #17) were either untreated or treated with Brefeldin A (BFA, 2 µg/ml) for 8h and protein lysates were prepared. Western blot was performed with total protein against the XBP1s antibody. Ponceau S staining or β-Actin is shown as a loading control.
Supplementary Figure 5

## Slide 6
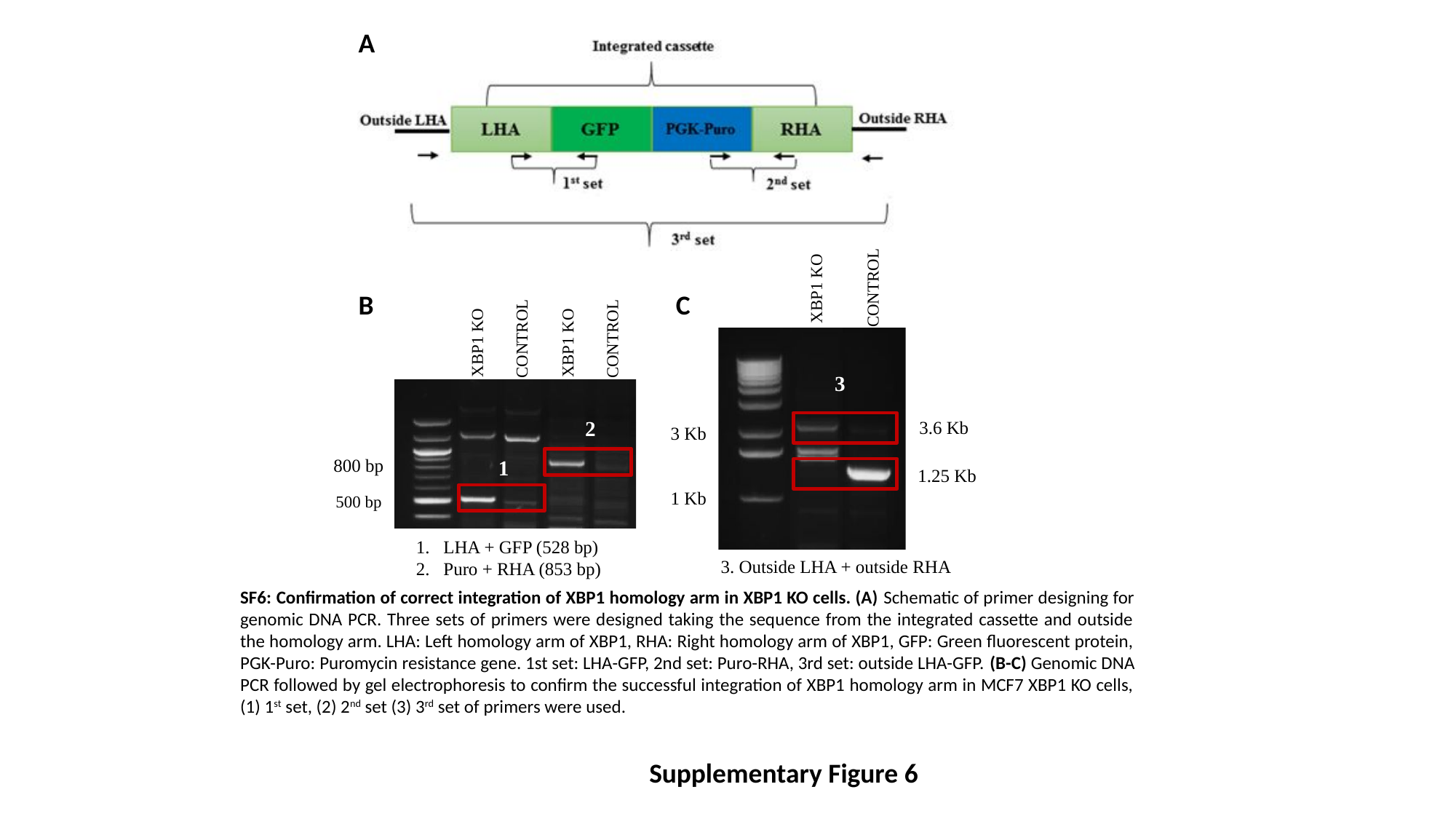

A
B C
CONTROL
XBP1 KO
3.6 Kb
3 Kb
1.25 Kb
1 Kb
3. Outside LHA + outside RHA
CONTROL
CONTROL
XBP1 KO
XBP1 KO
3
800 bp
500 bp
LHA + GFP (528 bp)
Puro + RHA (853 bp)
2
1
SF6: Confirmation of correct integration of XBP1 homology arm in XBP1 KO cells. (A) Schematic of primer designing for genomic DNA PCR. Three sets of primers were designed taking the sequence from the integrated cassette and outside the homology arm. LHA: Left homology arm of XBP1, RHA: Right homology arm of XBP1, GFP: Green fluorescent protein, PGK-Puro: Puromycin resistance gene. 1st set: LHA-GFP, 2nd set: Puro-RHA, 3rd set: outside LHA-GFP. (B-C) Genomic DNA PCR followed by gel electrophoresis to confirm the successful integration of XBP1 homology arm in MCF7 XBP1 KO cells, (1) 1st set, (2) 2nd set (3) 3rd set of primers were used.
Supplementary Figure 6

## Slide 7
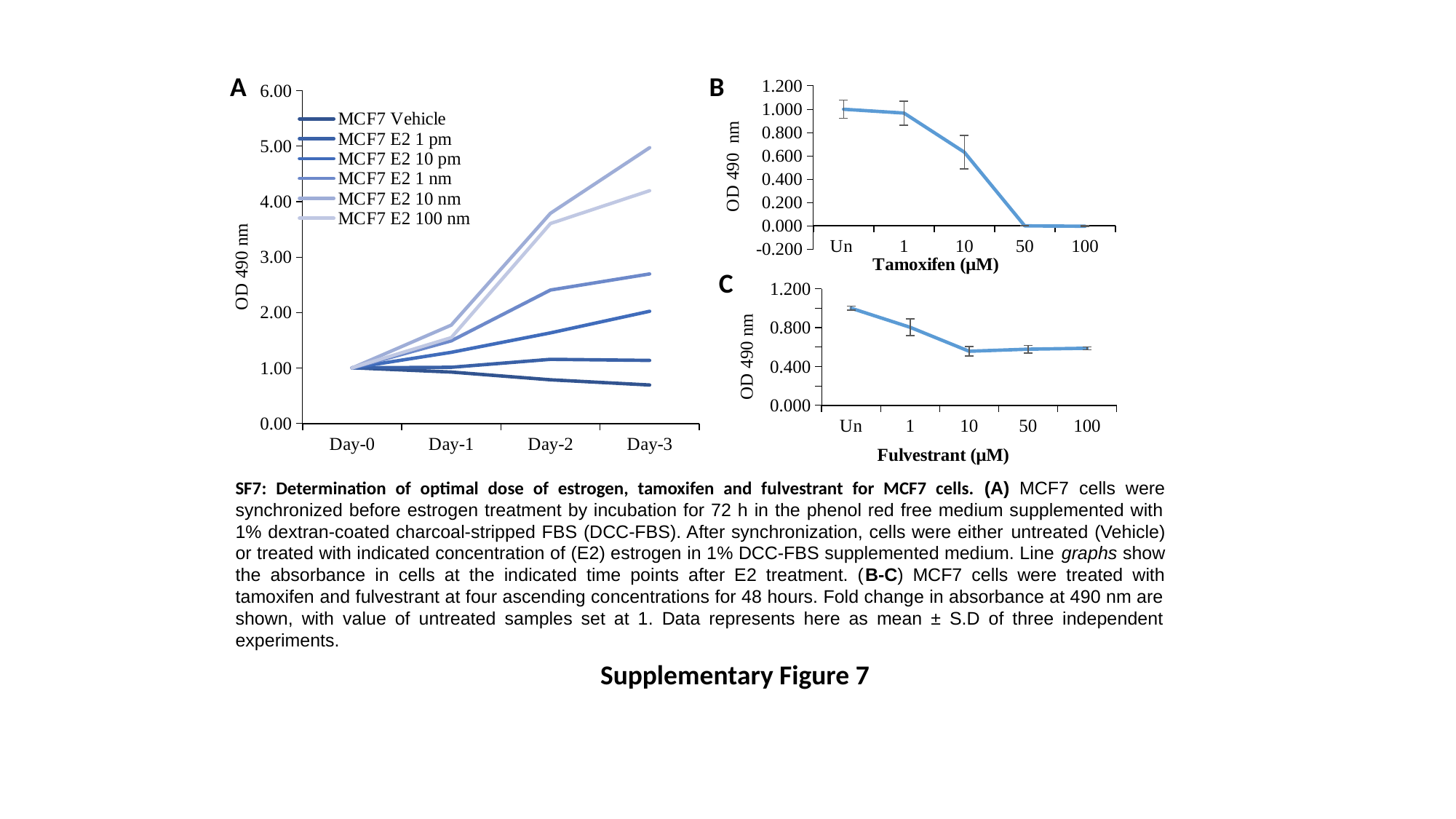

A B
 C
### Chart
| Category | MCF7 Vehicle | MCF7 E2 1 pm | MCF7 E2 10 pm | MCF7 E2 1 nm | MCF7 E2 10 nm | MCF7 E2 100 nm |
|---|---|---|---|---|---|---|
| Day-0 | 1.0045454545454544 | 1.003076923076923 | 1.0017241379310344 | 1.0 | 0.9999999999999999 | 1.0030769230769232 |
| Day-1 | 0.9272727272727277 | 1.012820512820513 | 1.2830459770114944 | 1.490049751243781 | 1.7733918128654973 | 1.5476923076923075 |
| Day-2 | 0.7878787878787875 | 1.1564102564102563 | 1.63275862068965 | 2.40547263681592 | 3.789473684210526 | 3.6051282051282043 |
| Day-3 | 0.693939393939394 | 1.1371794871794871 | 2.02313218390804 | 2.6955223880597 | 4.971929824561403 | 4.197435897435897 |
### Chart
| Category | MCF7 |
|---|---|
| Un | 1.0 |
| 1 | 0.967003959524857 |
| 10 | 0.6314708901598475 |
| 50 | -0.0004888302292613779 |
| 100 | -0.003519577650681921 |
### Chart
| Category | MCF7 |
|---|---|
| Un | 0.999999355462871 |
| 1 | 0.8032543968711587 |
| 10 | 0.55684772359154 |
| 50 | 0.578246807446466 |
| 100 | 0.587930977807942 |SF7: Determination of optimal dose of estrogen, tamoxifen and fulvestrant for MCF7 cells. (A) MCF7 cells were synchronized before estrogen treatment by incubation for 72 h in the phenol red free medium supplemented with 1% dextran-coated charcoal-stripped FBS (DCC-FBS). After synchronization, cells were either untreated (Vehicle) or treated with indicated concentration of (E2) estrogen in 1% DCC-FBS supplemented medium. Line graphs show the absorbance in cells at the indicated time points after E2 treatment. (B-C) MCF7 cells were treated with tamoxifen and fulvestrant at four ascending concentrations for 48 hours. Fold change in absorbance at 490 nm are shown, with value of untreated samples set at 1. Data represents here as mean ± S.D of three independent experiments.
Supplementary Figure 7

## Slide 8
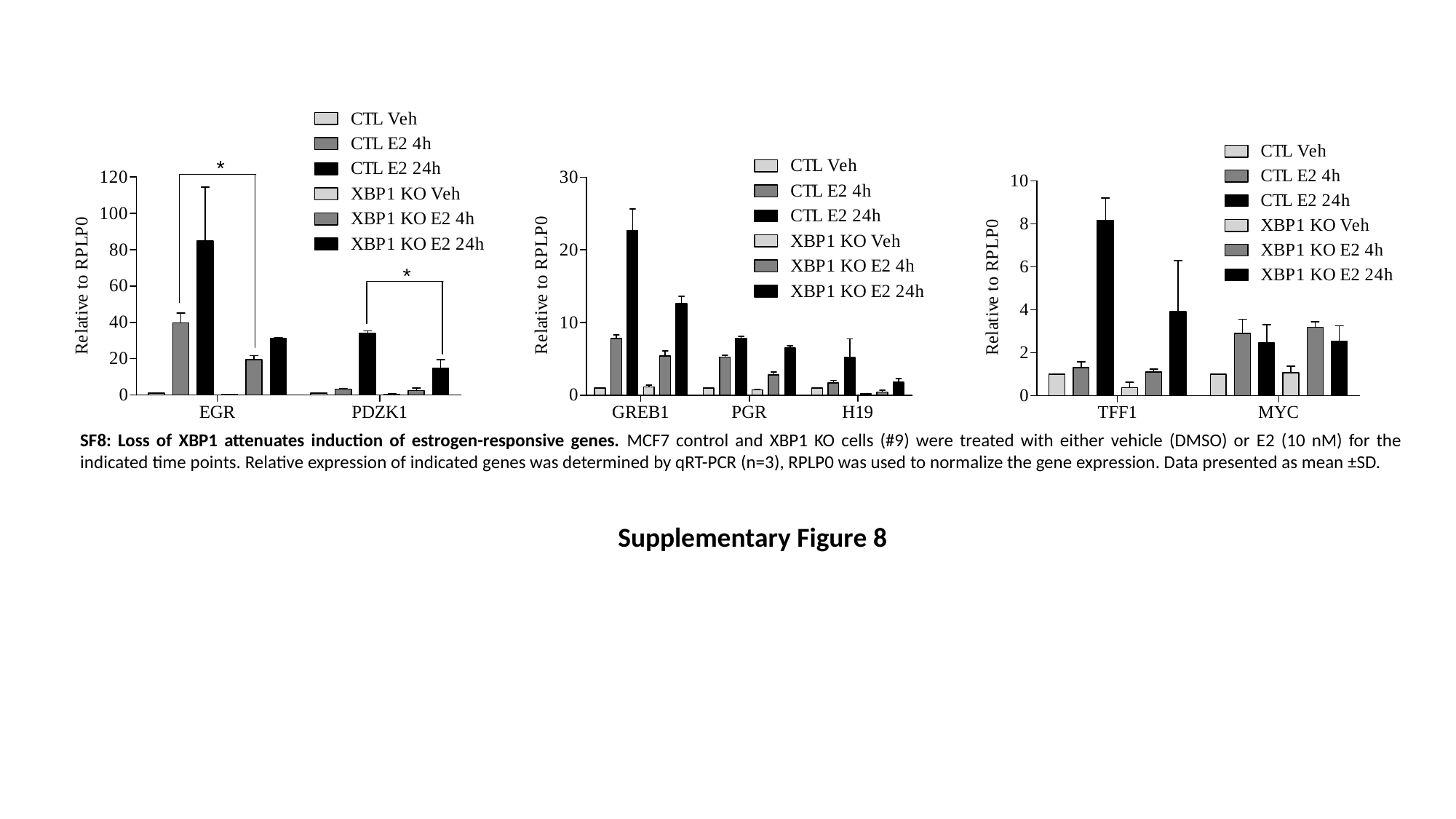

SF8: Loss of XBP1 attenuates induction of estrogen-responsive genes. MCF7 control and XBP1 KO cells (#9) were treated with either vehicle (DMSO) or E2 (10 nM) for the indicated time points. Relative expression of indicated genes was determined by qRT-PCR (n=3), RPLP0 was used to normalize the gene expression. Data presented as mean ±SD.
Supplementary Figure 8

## Slide 9
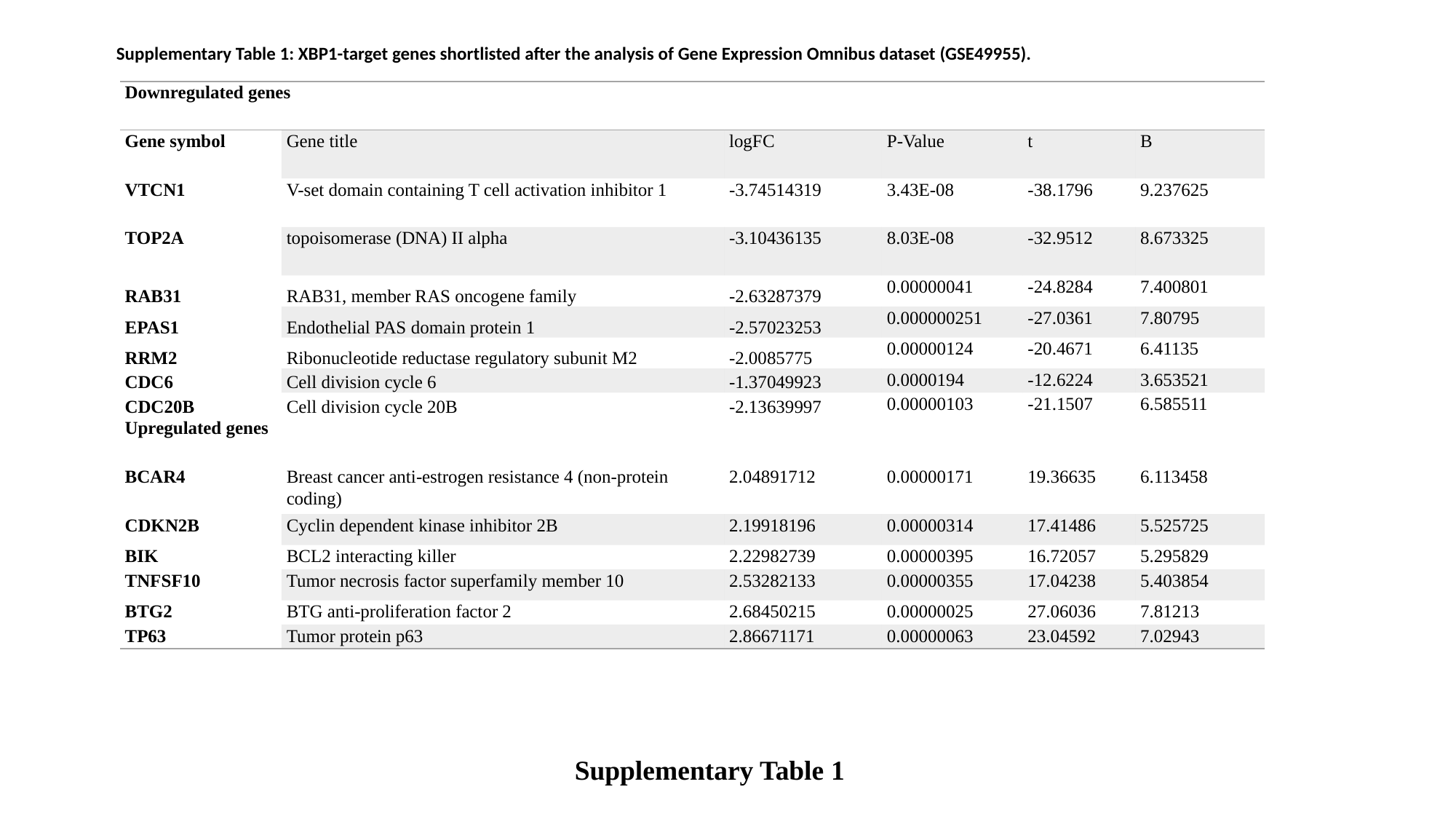

Supplementary Table 1: XBP1-target genes shortlisted after the analysis of Gene Expression Omnibus dataset (GSE49955).
| Downregulated genes | | | | | |
| --- | --- | --- | --- | --- | --- |
| Gene symbol | Gene title | logFC | P-Value | t | B |
| VTCN1 | V-set domain containing T cell activation inhibitor 1 | -3.74514319 | 3.43E-08 | -38.1796 | 9.237625 |
| TOP2A | topoisomerase (DNA) II alpha | -3.10436135 | 8.03E-08 | -32.9512 | 8.673325 |
| RAB31 | RAB31, member RAS oncogene family | -2.63287379 | 0.00000041 | -24.8284 | 7.400801 |
| EPAS1 | Endothelial PAS domain protein 1 | -2.57023253 | 0.000000251 | -27.0361 | 7.80795 |
| RRM2 | Ribonucleotide reductase regulatory subunit M2 | -2.0085775 | 0.00000124 | -20.4671 | 6.41135 |
| CDC6 | Cell division cycle 6 | -1.37049923 | 0.0000194 | -12.6224 | 3.653521 |
| CDC20B | Cell division cycle 20B | -2.13639997 | 0.00000103 | -21.1507 | 6.585511 |
| Upregulated genes | | | | | |
| BCAR4 | Breast cancer anti-estrogen resistance 4 (non-protein coding) | 2.04891712 | 0.00000171 | 19.36635 | 6.113458 |
| CDKN2B | Cyclin dependent kinase inhibitor 2B | 2.19918196 | 0.00000314 | 17.41486 | 5.525725 |
| BIK | BCL2 interacting killer | 2.22982739 | 0.00000395 | 16.72057 | 5.295829 |
| TNFSF10 | Tumor necrosis factor superfamily member 10 | 2.53282133 | 0.00000355 | 17.04238 | 5.403854 |
| BTG2 | BTG anti-proliferation factor 2 | 2.68450215 | 0.00000025 | 27.06036 | 7.81213 |
| TP63 | Tumor protein p63 | 2.86671171 | 0.00000063 | 23.04592 | 7.02943 |
Supplementary Table 1

## Slide 10
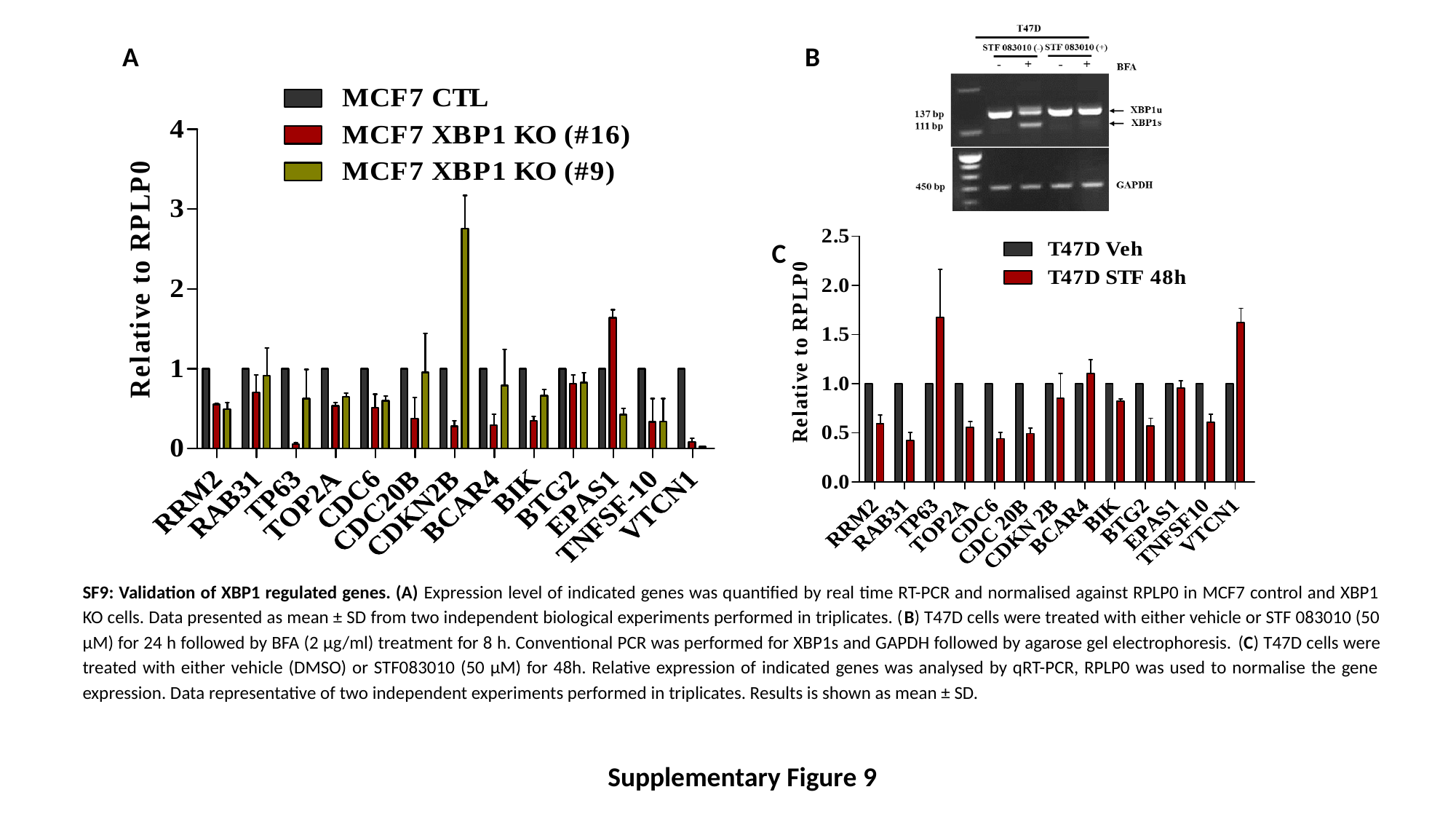

A B
 C
SF9: Validation of XBP1 regulated genes. (A) Expression level of indicated genes was quantified by real time RT-PCR and normalised against RPLP0 in MCF7 control and XBP1 KO cells. Data presented as mean ± SD from two independent biological experiments performed in triplicates. (B) T47D cells were treated with either vehicle or STF 083010 (50 µM) for 24 h followed by BFA (2 µg/ml) treatment for 8 h. Conventional PCR was performed for XBP1s and GAPDH followed by agarose gel electrophoresis. (C) T47D cells were treated with either vehicle (DMSO) or STF083010 (50 µM) for 48h. Relative expression of indicated genes was analysed by qRT-PCR, RPLP0 was used to normalise the gene expression. Data representative of two independent experiments performed in triplicates. Results is shown as mean ± SD.
Supplementary Figure 9

## Slide 11
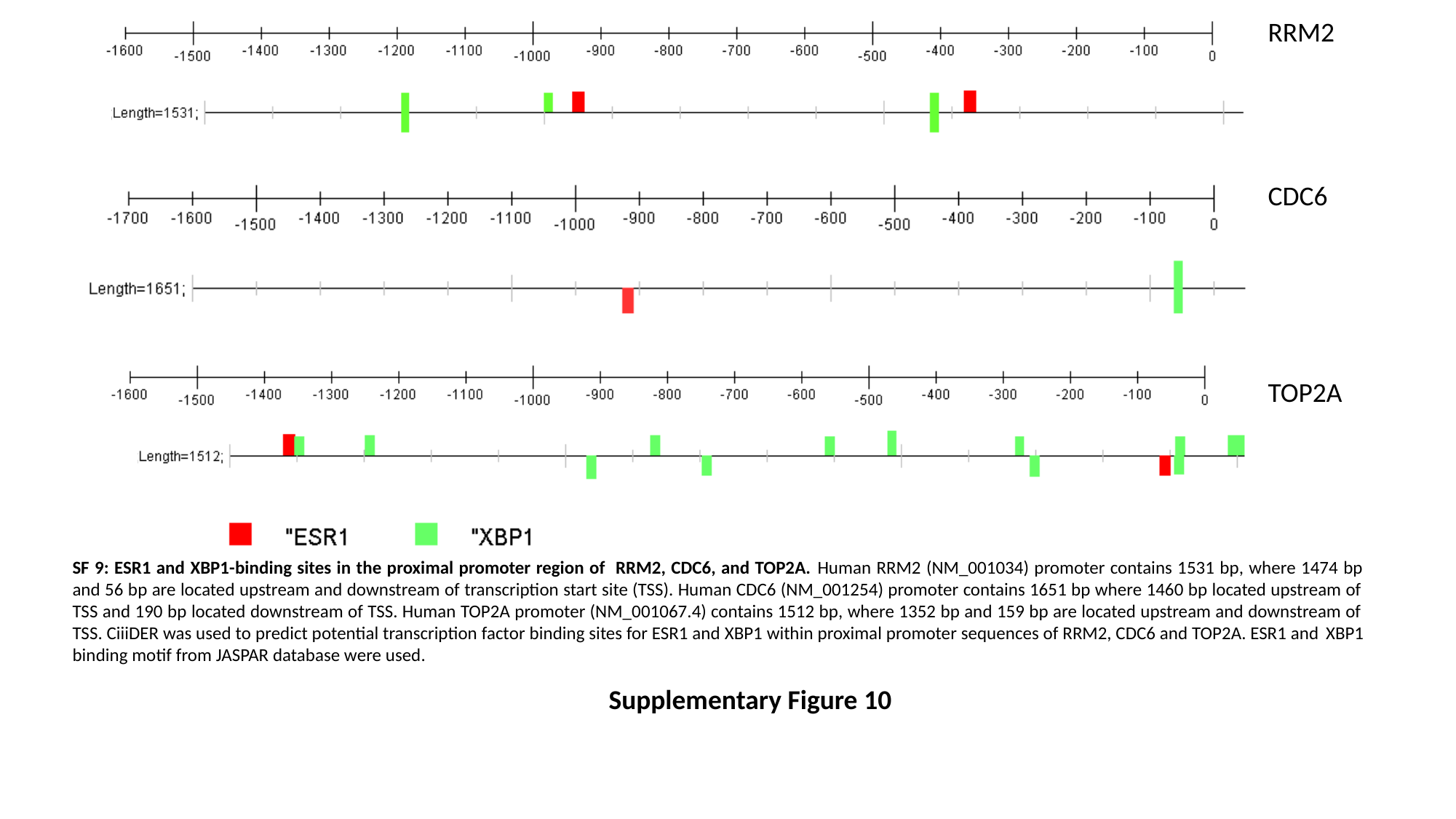

RRM2
CDC6
TOP2A
SF 9: ESR1 and XBP1-binding sites in the proximal promoter region of RRM2, CDC6, and TOP2A. Human RRM2 (NM_001034) promoter contains 1531 bp, where 1474 bp and 56 bp are located upstream and downstream of transcription start site (TSS). Human CDC6 (NM_001254) promoter contains 1651 bp where 1460 bp located upstream of TSS and 190 bp located downstream of TSS. Human TOP2A promoter (NM_001067.4) contains 1512 bp, where 1352 bp and 159 bp are located upstream and downstream of TSS. CiiiDER was used to predict potential transcription factor binding sites for ESR1 and XBP1 within proximal promoter sequences of RRM2, CDC6 and TOP2A. ESR1 and XBP1 binding motif from JASPAR database were used.
Supplementary Figure 10

## Slide 12
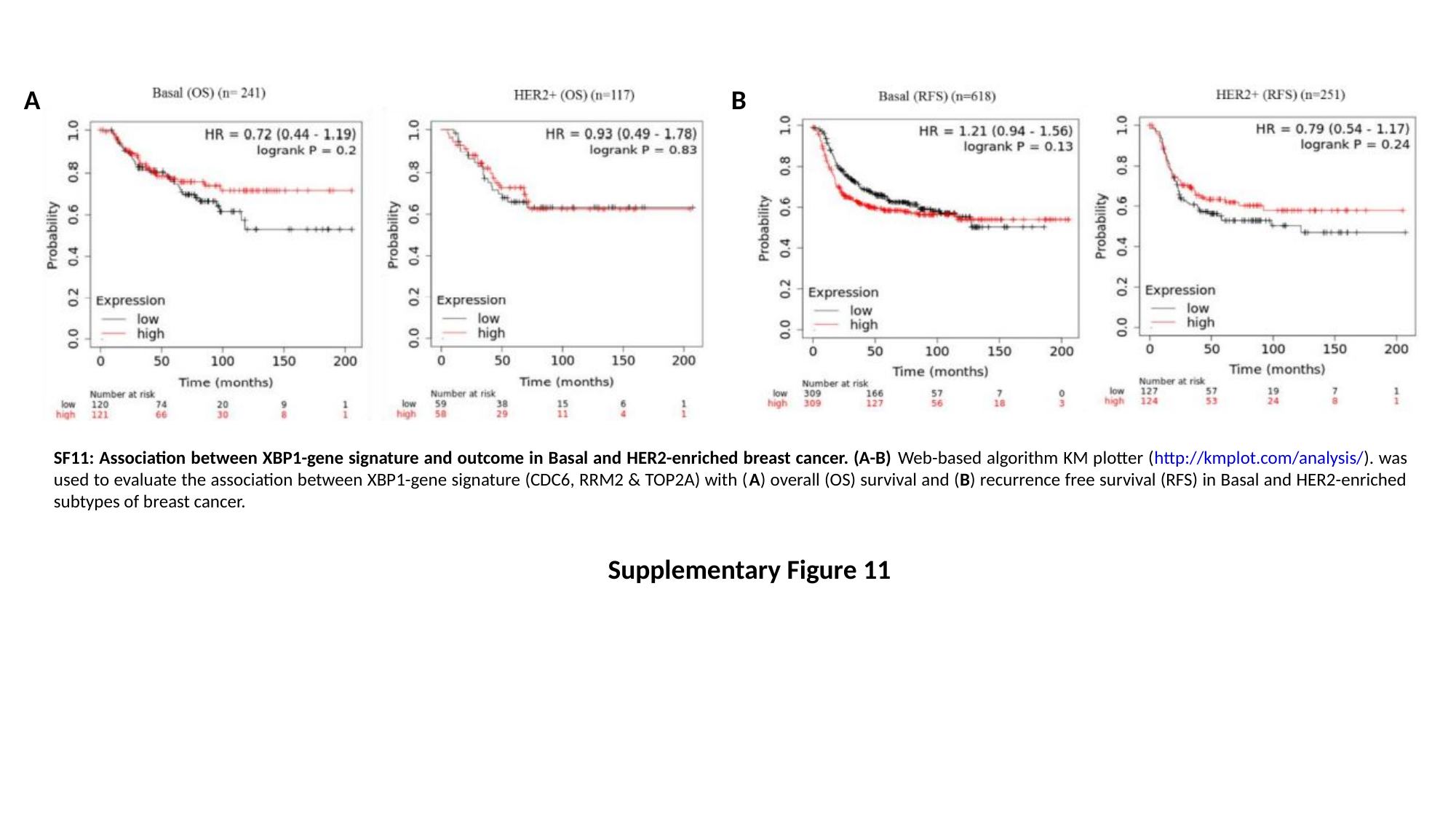

SF11: Association between XBP1-gene signature and outcome in Basal and HER2-enriched breast cancer. (A-B) Web-based algorithm KM plotter (http://kmplot.com/analysis/). was used to evaluate the association between XBP1-gene signature (CDC6, RRM2 & TOP2A) with (A) overall (OS) survival and (B) recurrence free survival (RFS) in Basal and HER2-enriched subtypes of breast cancer.
A B
Supplementary Figure 11

## Slide 13
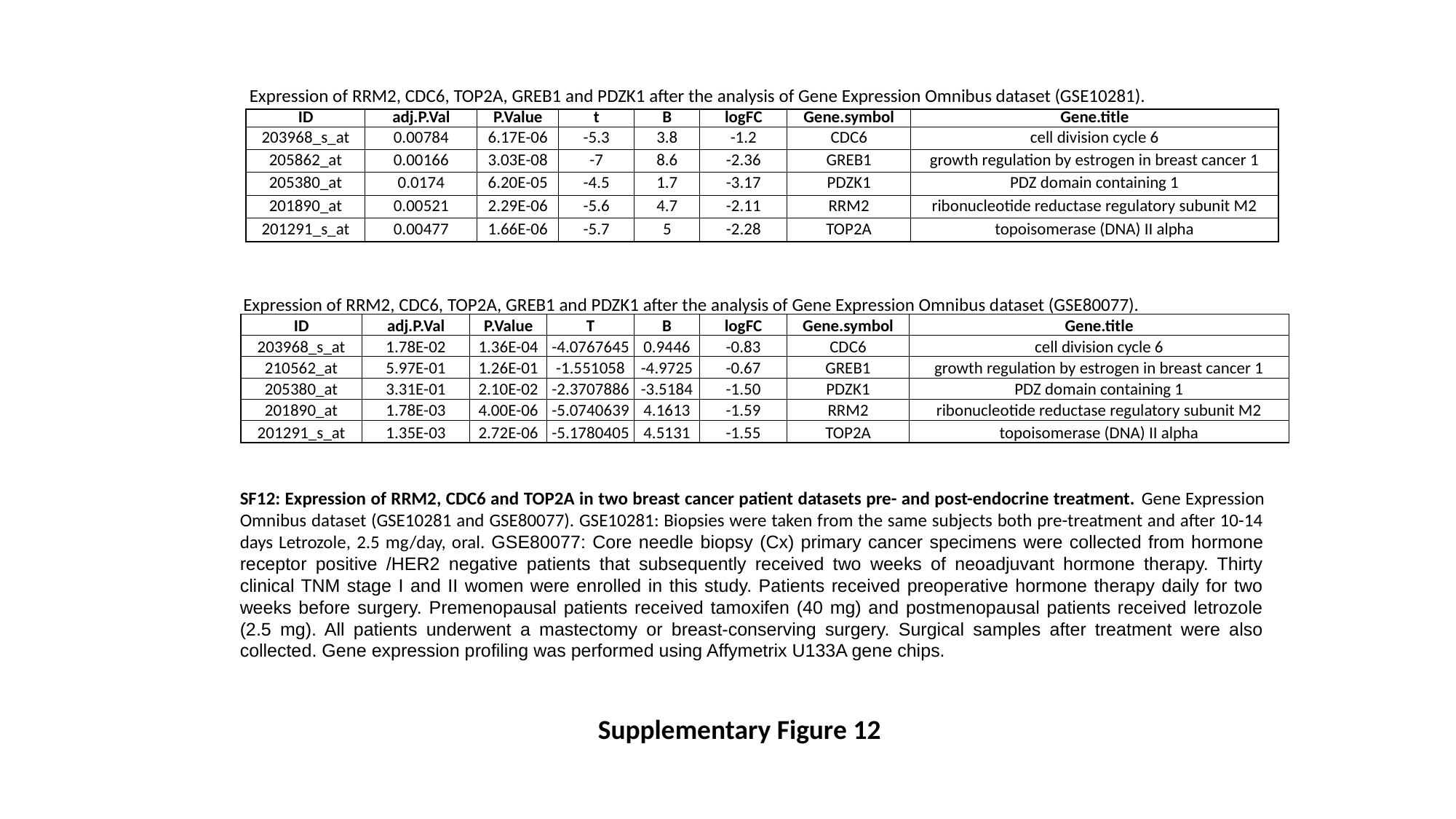

Expression of RRM2, CDC6, TOP2A, GREB1 and PDZK1 after the analysis of Gene Expression Omnibus dataset (GSE10281).
| ID | adj.P.Val | P.Value | t | B | logFC | Gene.symbol | Gene.title |
| --- | --- | --- | --- | --- | --- | --- | --- |
| 203968\_s\_at | 0.00784 | 6.17E-06 | -5.3 | 3.8 | -1.2 | CDC6 | cell division cycle 6 |
| 205862\_at | 0.00166 | 3.03E-08 | -7 | 8.6 | -2.36 | GREB1 | growth regulation by estrogen in breast cancer 1 |
| 205380\_at | 0.0174 | 6.20E-05 | -4.5 | 1.7 | -3.17 | PDZK1 | PDZ domain containing 1 |
| 201890\_at | 0.00521 | 2.29E-06 | -5.6 | 4.7 | -2.11 | RRM2 | ribonucleotide reductase regulatory subunit M2 |
| 201291\_s\_at | 0.00477 | 1.66E-06 | -5.7 | 5 | -2.28 | TOP2A | topoisomerase (DNA) II alpha |
Expression of RRM2, CDC6, TOP2A, GREB1 and PDZK1 after the analysis of Gene Expression Omnibus dataset (GSE80077).
| ID | adj.P.Val | P.Value | T | B | logFC | Gene.symbol | Gene.title |
| --- | --- | --- | --- | --- | --- | --- | --- |
| 203968\_s\_at | 1.78E-02 | 1.36E-04 | -4.0767645 | 0.9446 | -0.83 | CDC6 | cell division cycle 6 |
| 210562\_at | 5.97E-01 | 1.26E-01 | -1.551058 | -4.9725 | -0.67 | GREB1 | growth regulation by estrogen in breast cancer 1 |
| 205380\_at | 3.31E-01 | 2.10E-02 | -2.3707886 | -3.5184 | -1.50 | PDZK1 | PDZ domain containing 1 |
| 201890\_at | 1.78E-03 | 4.00E-06 | -5.0740639 | 4.1613 | -1.59 | RRM2 | ribonucleotide reductase regulatory subunit M2 |
| 201291\_s\_at | 1.35E-03 | 2.72E-06 | -5.1780405 | 4.5131 | -1.55 | TOP2A | topoisomerase (DNA) II alpha |
SF12: Expression of RRM2, CDC6 and TOP2A in two breast cancer patient datasets pre- and post-endocrine treatment. Gene Expression Omnibus dataset (GSE10281 and GSE80077). GSE10281: Biopsies were taken from the same subjects both pre-treatment and after 10-14 days Letrozole, 2.5 mg/day, oral. GSE80077: Core needle biopsy (Cx) primary cancer specimens were collected from hormone receptor positive /HER2 negative patients that subsequently received two weeks of neoadjuvant hormone therapy. Thirty clinical TNM stage I and II women were enrolled in this study. Patients received preoperative hormone therapy daily for two weeks before surgery. Premenopausal patients received tamoxifen (40 mg) and postmenopausal patients received letrozole (2.5 mg). All patients underwent a mastectomy or breast-conserving surgery. Surgical samples after treatment were also collected. Gene expression profiling was performed using Affymetrix U133A gene chips.
Supplementary Figure 12
