# Supplementary material for: RRM2 and CDC6 are novel effectors of XBP1-mediated endocrine resistance and predictive markers of tamoxifen sensitivity
Source: BMC Cancer. 2023 Mar 30;23:288. doi: 10.1186/s12885-023-10745-1 (PMC10061897; doi:10.1186/s12885-023-10745-1)
Supplement: Supplementary file 2 — Additional file 2. [file 12885_2023_10745_MOESM2_ESM.pdf]

Uncropped gel for Figure 1 (A)

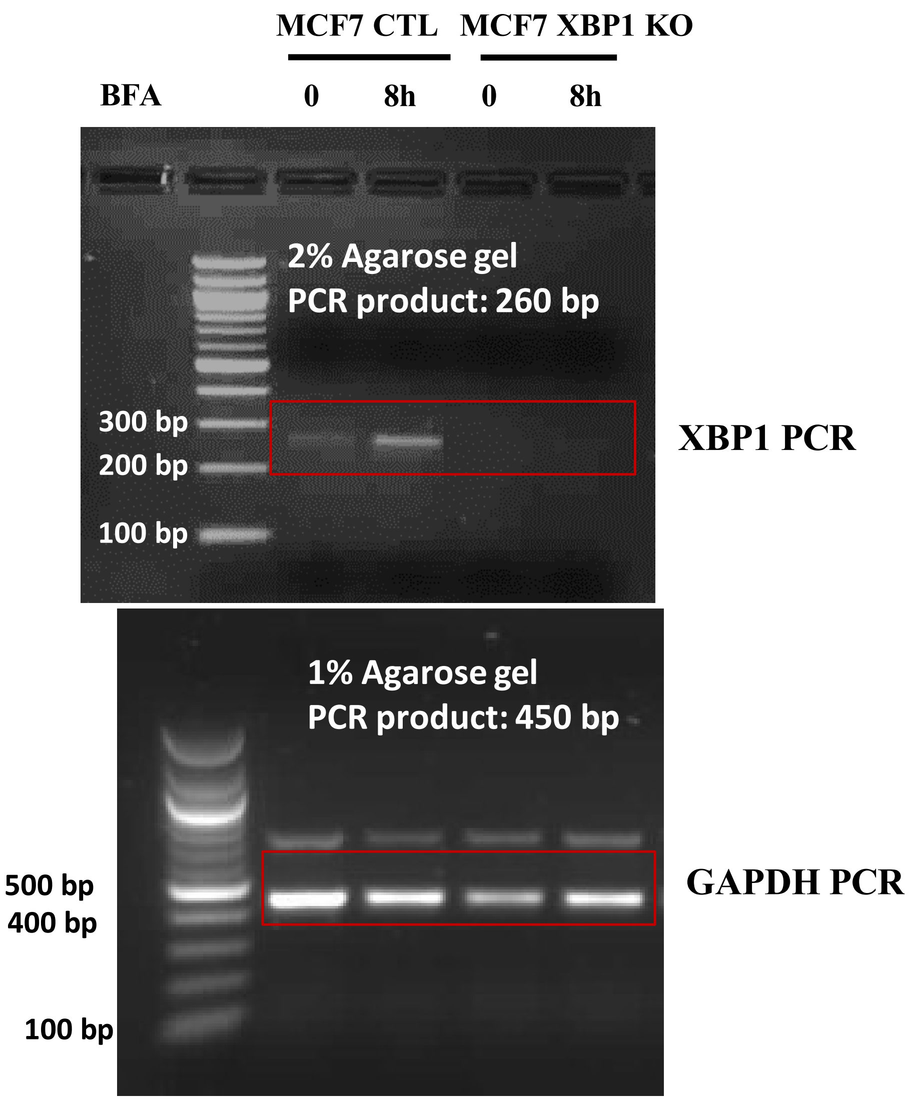

Uncropped blot for Figure 1 (B)

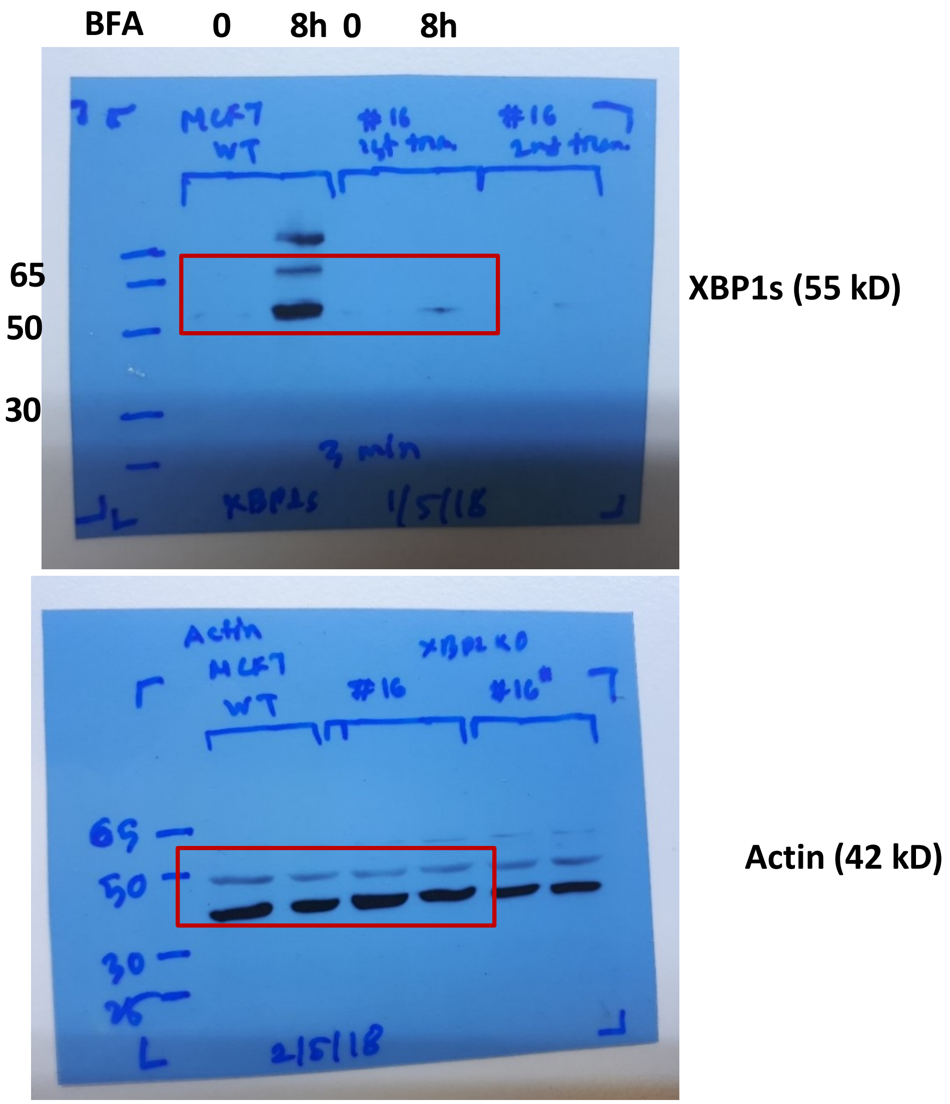

Uncropped blot for Figure 3 (B)

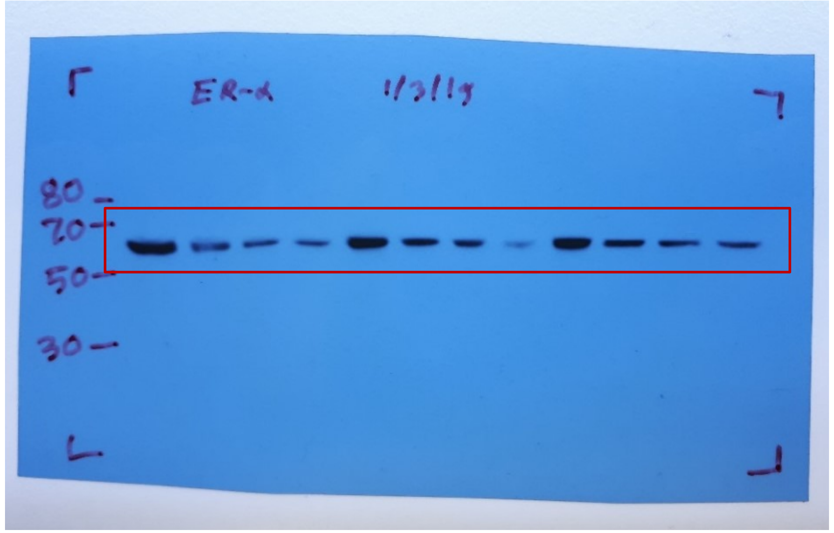

ER-*α* (66 kD)

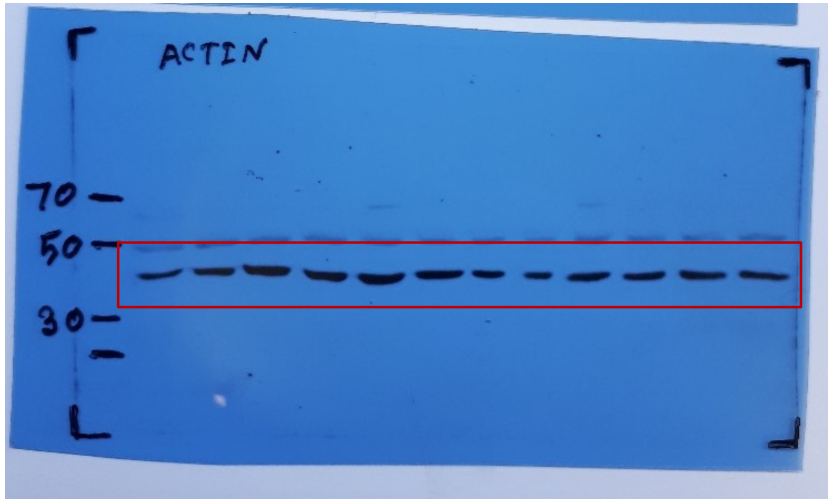

Actin (42 kD)

Uncropped blot for Figure 4 (A)

1. MCF7 CTL
2. MCF7 XBP1 KO (#16)
3. MCF7 XBP1 KO (#9)

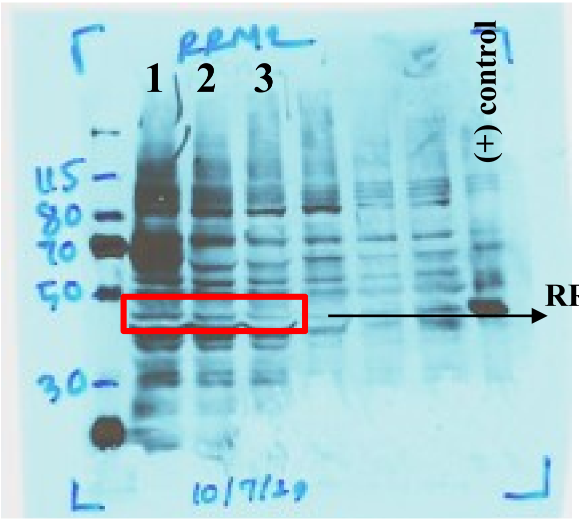

RRM2 (45 kD)

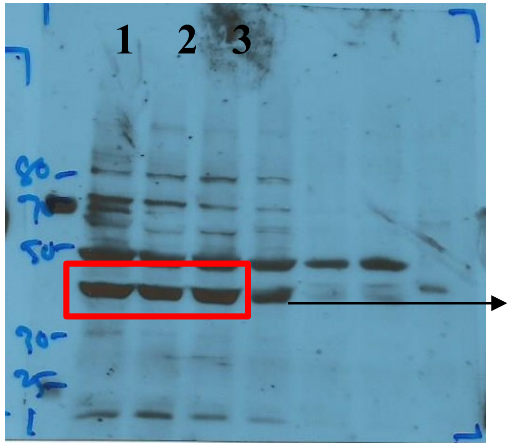

Actin (42 kD)

1. MCF7 CTL
2. MCF7 XBP1 KO (#16)
3. MCF7 XBP1 KO (#9)

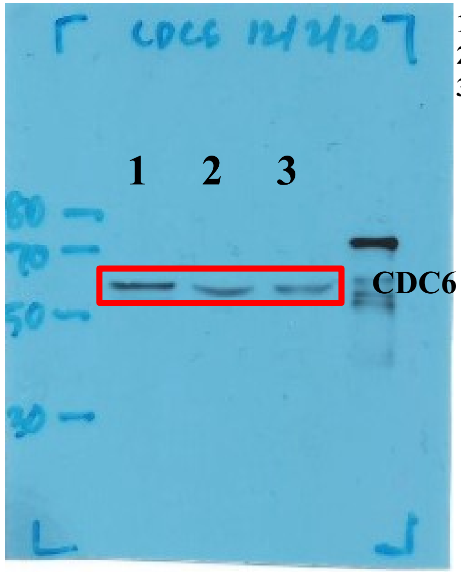

CDC6 (62 kD)

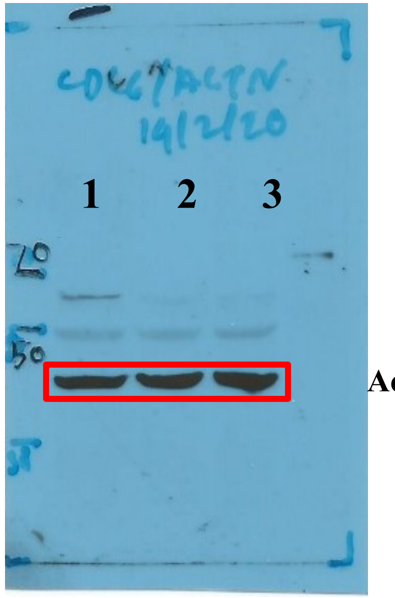

Actin (42 kD)

1. MCF7 CTL
2. MCF7 XBP1 KO (#16)
3. MCF7 XBP1 KO (#9)

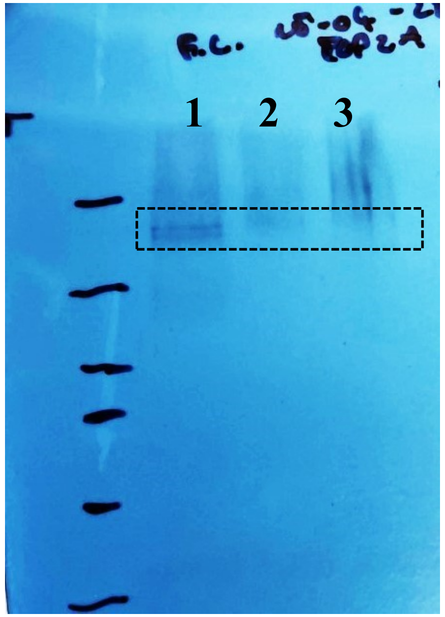

TOP2A (174 kD)

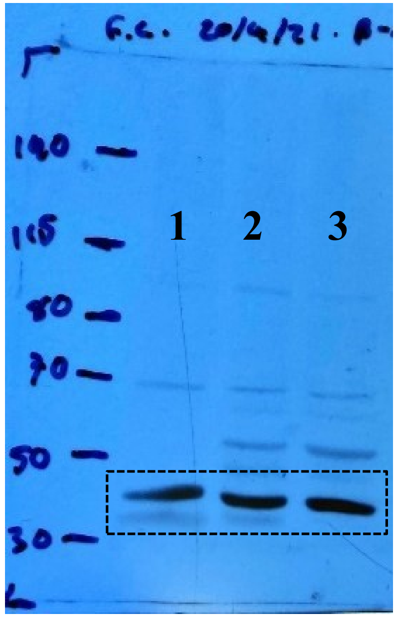

Actin (42 kD)

Uncropped blot for Figure 4 (B)

1. MCF7 VC
2. MCF7 STF083010

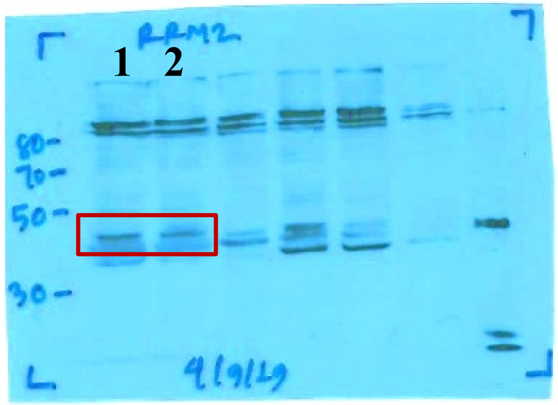

RRM2 (45 kD)

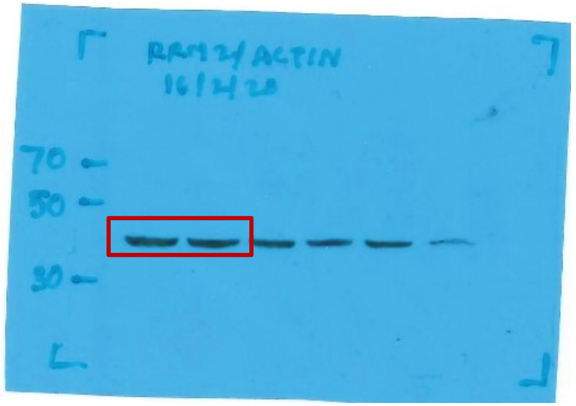

Actin (42 kD)

1. MCF7 VC
2. MCF7 STF083010

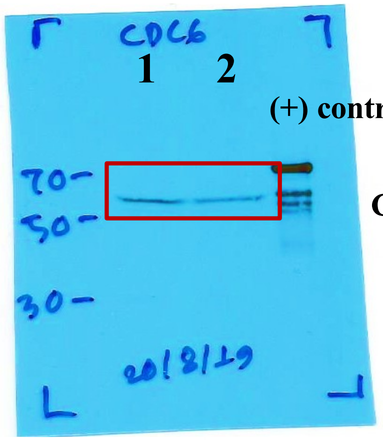

CDC6 (62 kD)

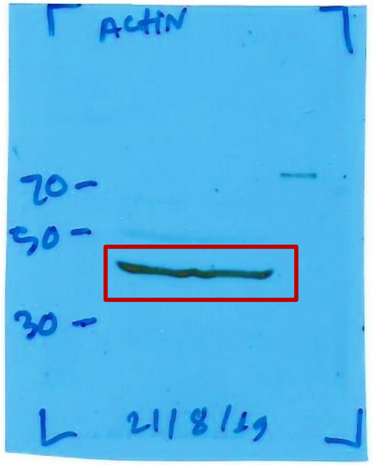

Actin

1. MCF7 VC
2. MCF7 STF083010

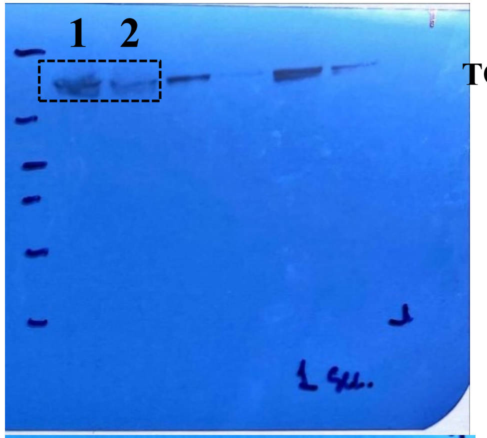

TOP2A (174 kD)

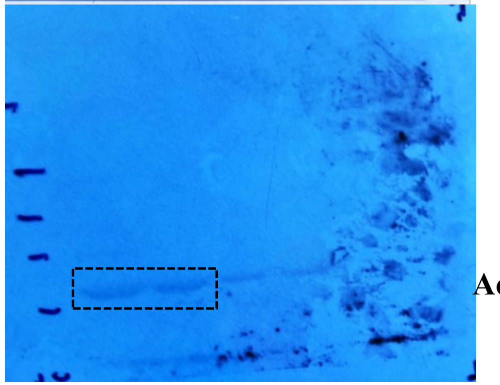

Actin (42 kD)

Uncropped blot for Figure 4 (C)

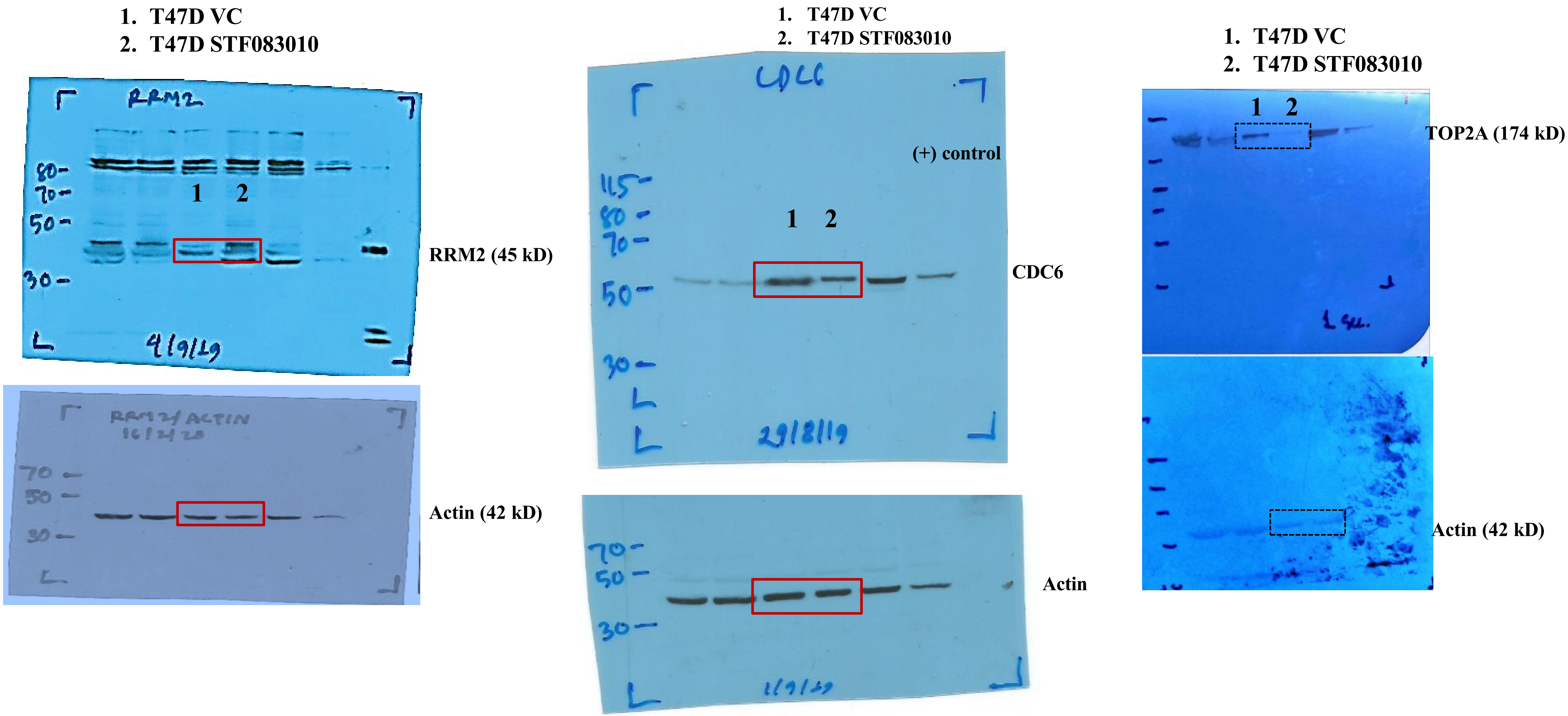

Uncropped blot for Figure 4 (D)

1. BT474 VC
2. BT474 STF083010

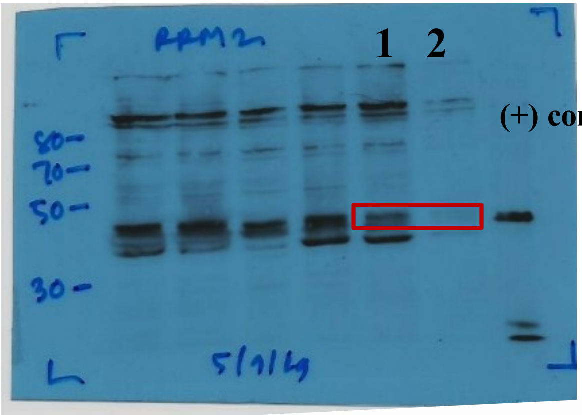

(+) control

RRM2 (45 kD)

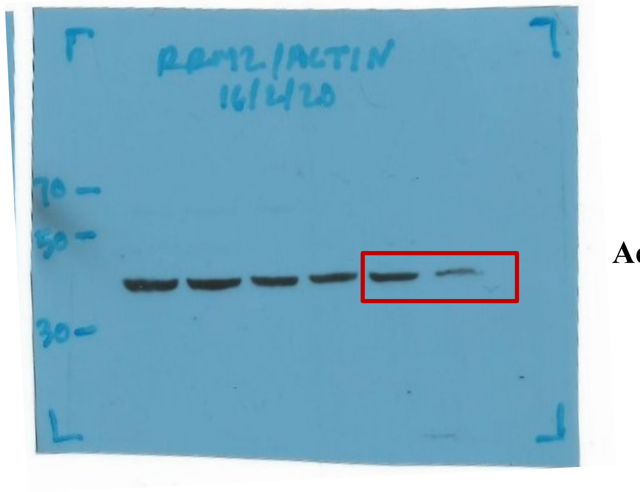

Actin (42 kD)

1. BT474 VC
2. BT474 STF083010

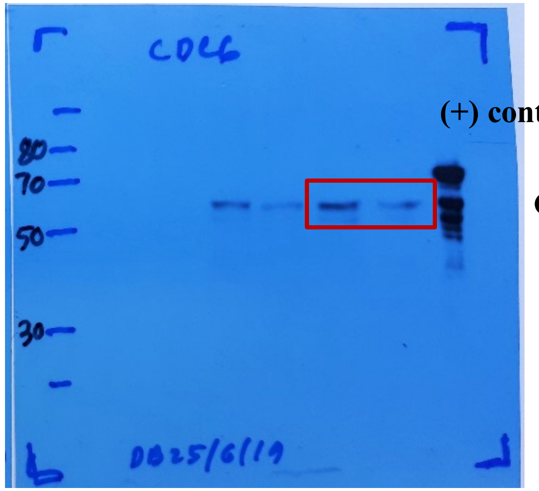

(+) control

CDC6 (62 kD)

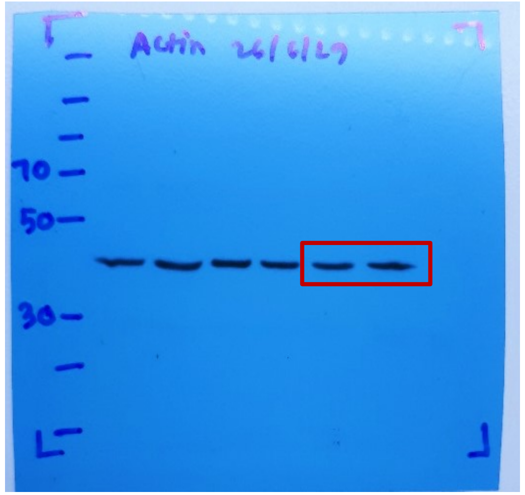

Actin (42 kD)

1. BT474 VC
2. BT474 STF083010

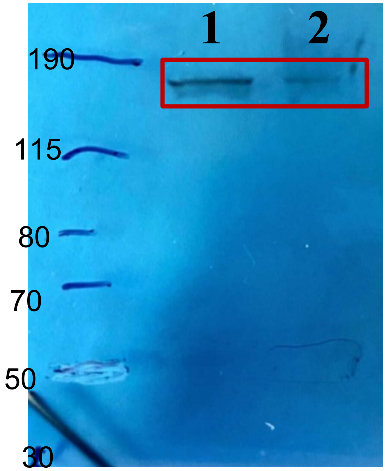

TOP2A (174 kD)

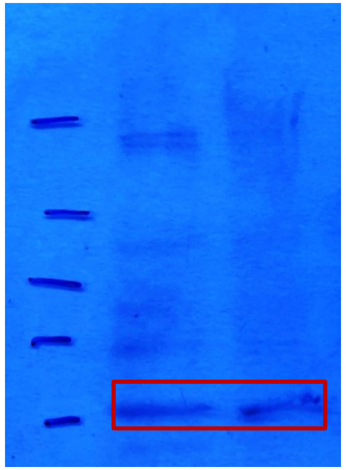

Actin (42 kD)

Uncropped blot for Figure 6 (A)

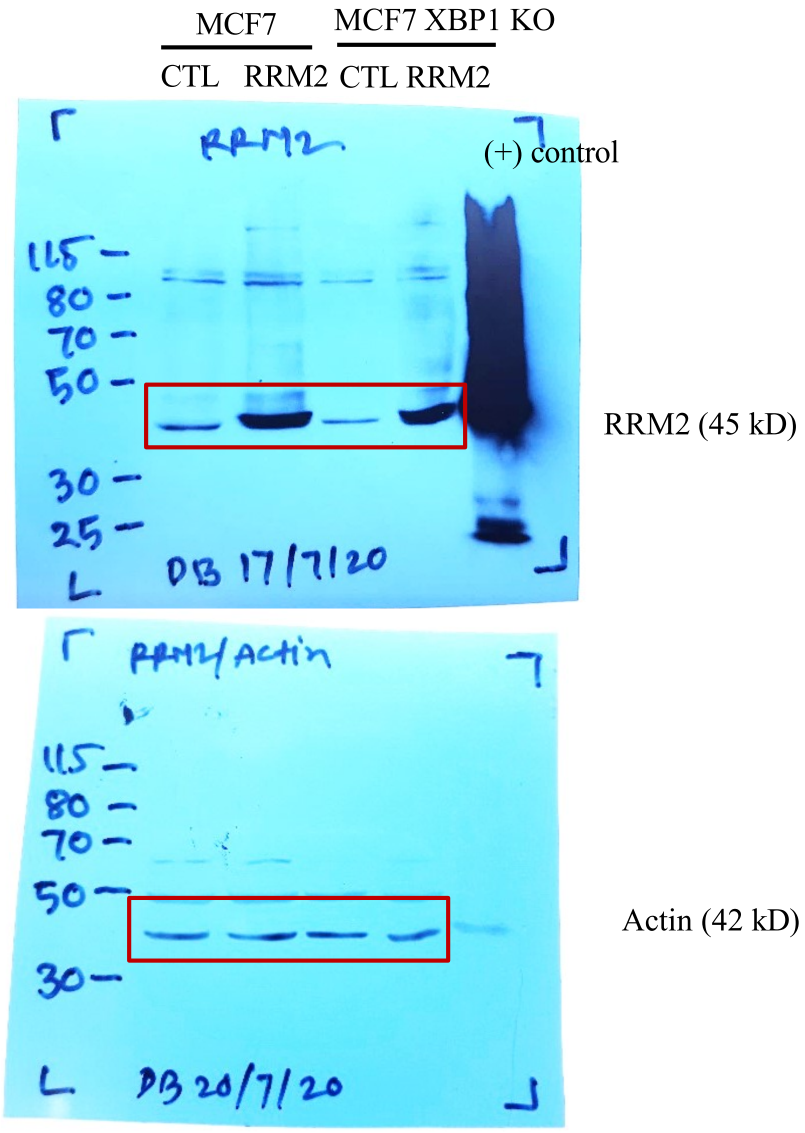

Uncropped blot for Figure 6 (C)

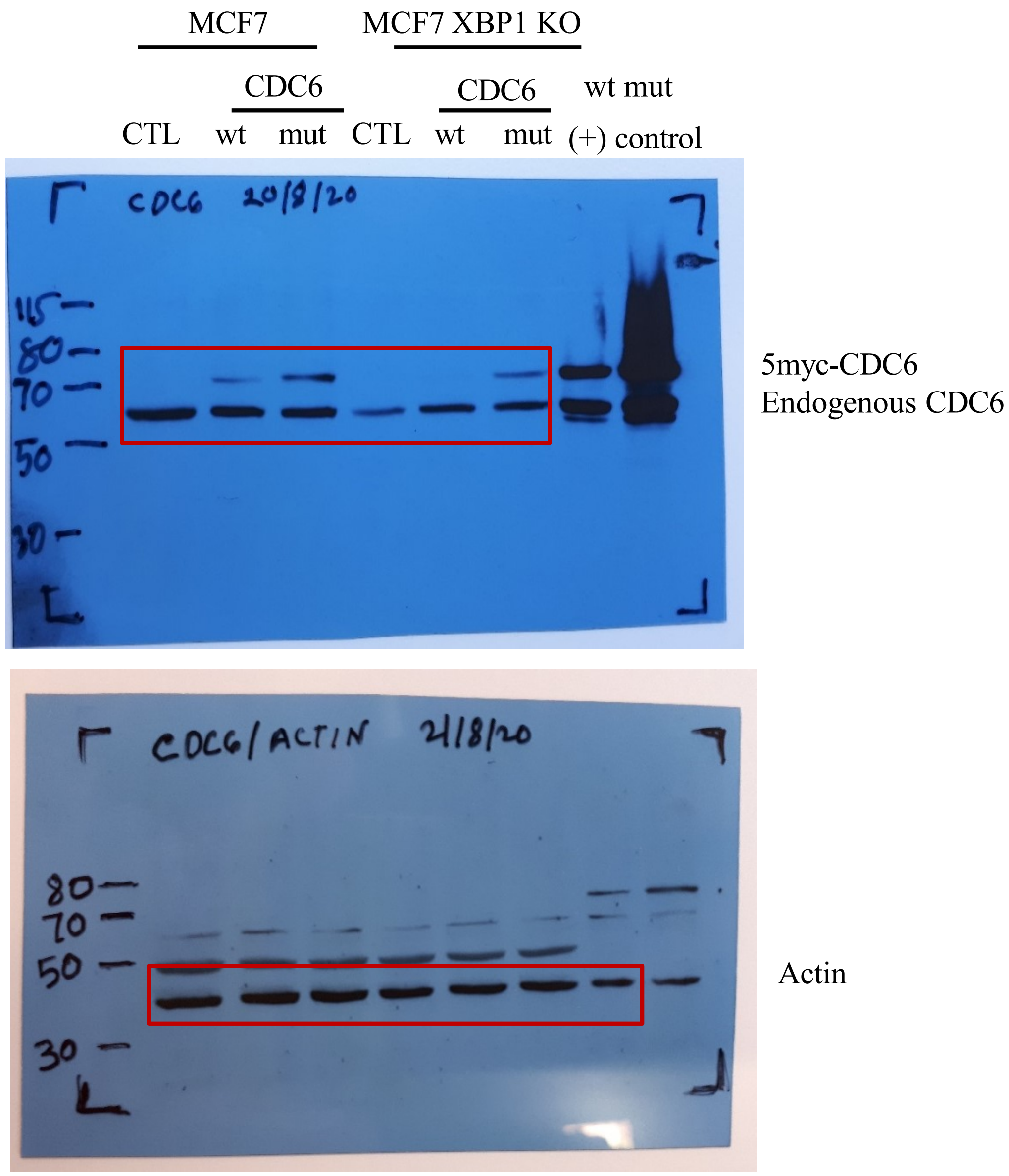

Uncropped blots for Supplementary Figure 5

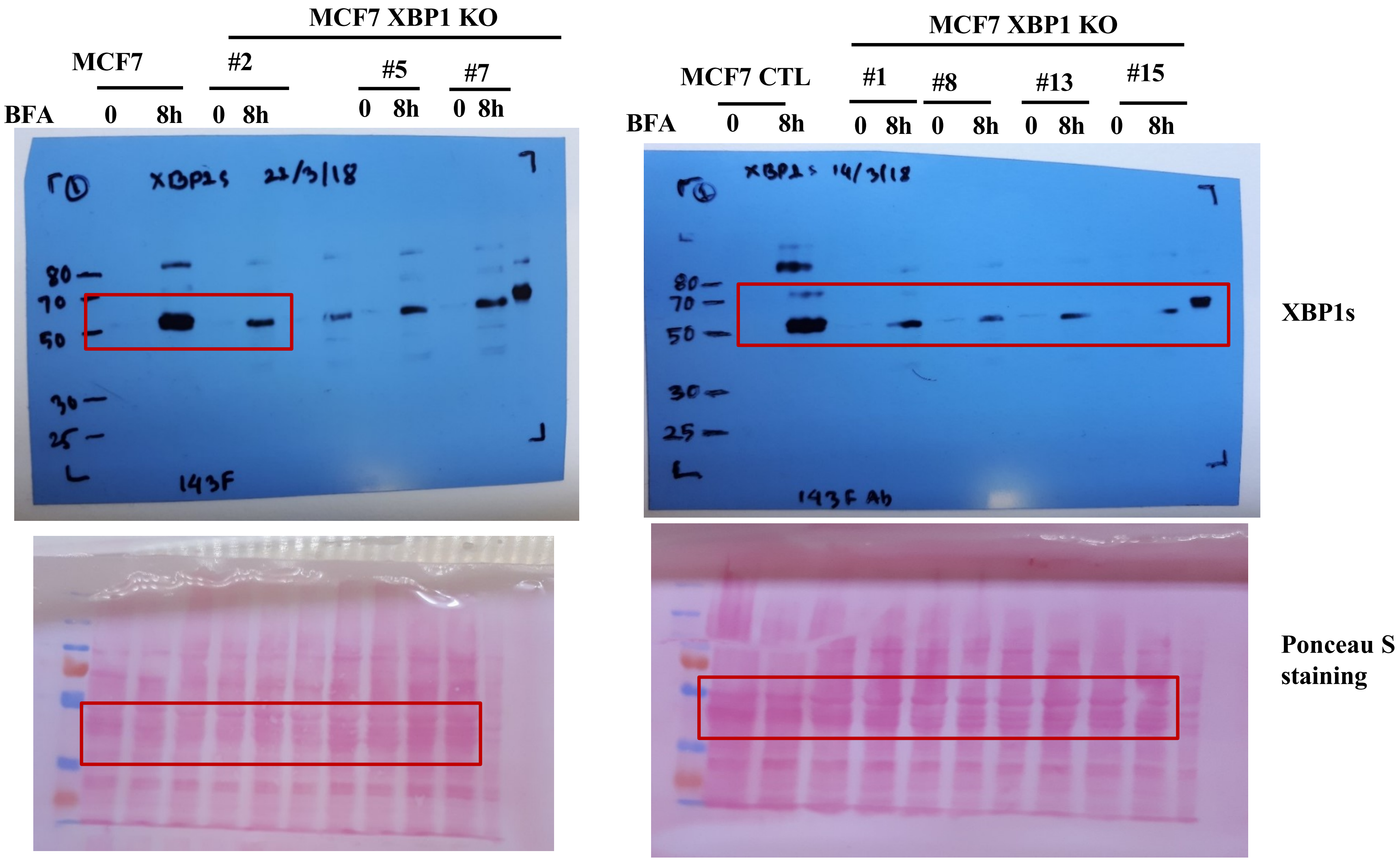

Uncropped blots for Supplementary Figure 5

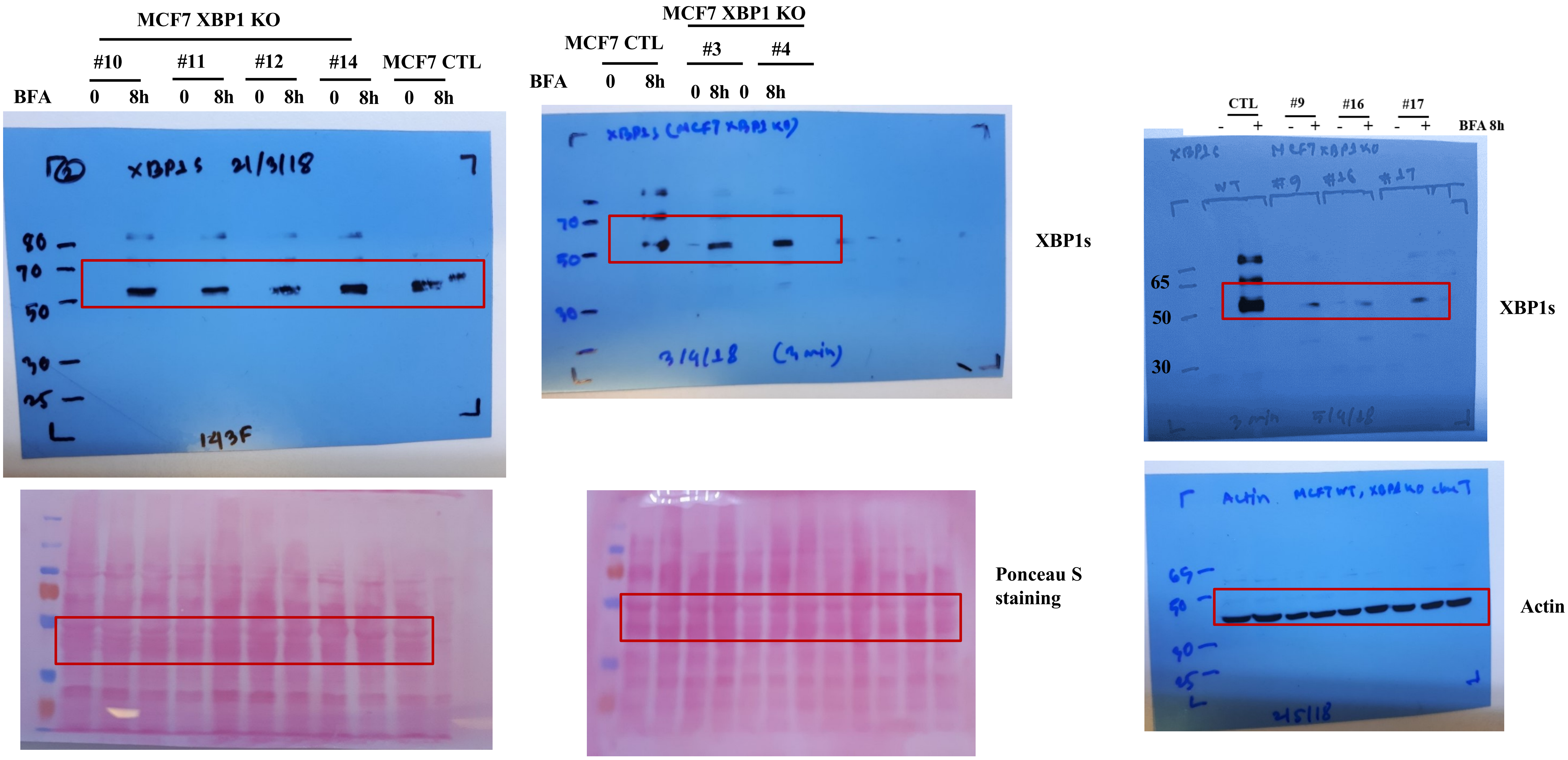

Full gel image for SF6 and SF8

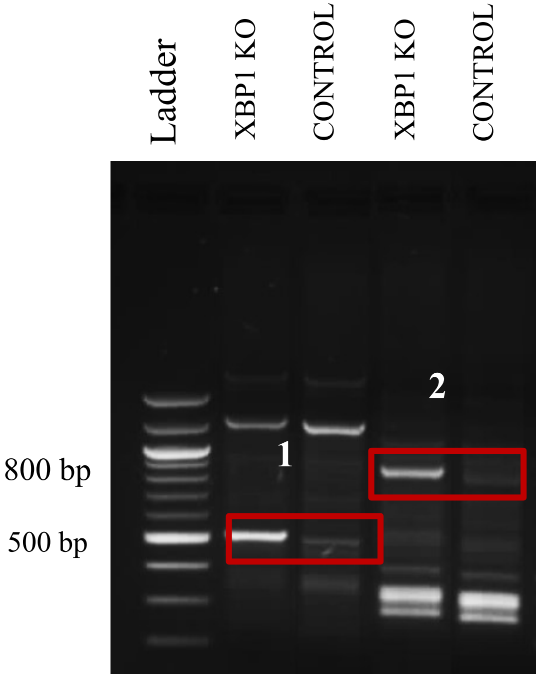

1. LHA + GFP (528 bp)
2. Puro + RHA (853 bp)

SF6 (B)

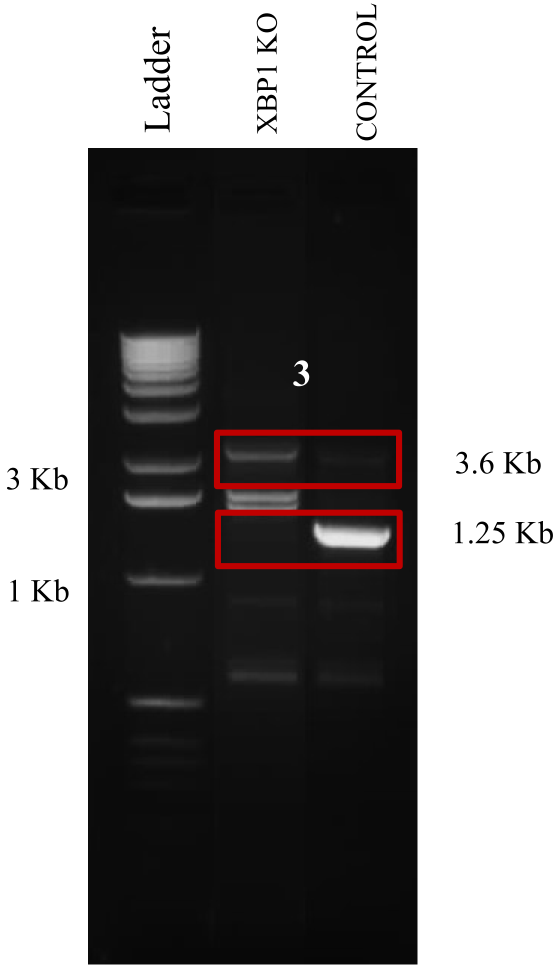

3. Outside LHA + outside RHA

SF6 (C)

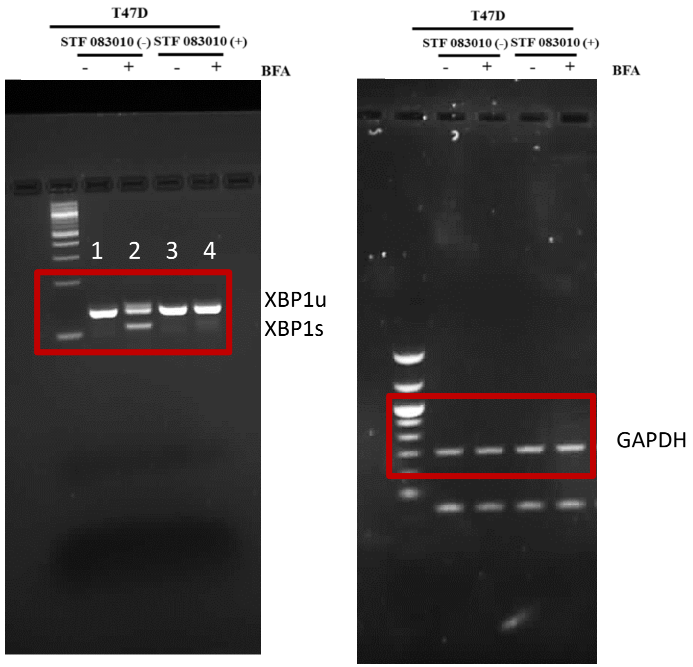

SF8 (B)
